# Rationale and design of the 2 by 2 factorial design GnG-trial: a randomized phase-III study to compare two schedules of gemtuzumab ozogamicin as adjunct to intensive induction therapy and to compare double-blinded intensive postremission therapy with or without glasdegib in older patients with newly diagnosed AML

**DOI:** 10.1186/s13063-021-05703-w

**Published:** 2021-11-03

**Authors:** Sonia Jaramillo, Johannes Krisam, Lucian Le Cornet, Markus Kratzmann, Lukas Baumann, Tim Sauer, Martina Crysandt, Andreas Rank, Dirk Behringer, Lino Teichmann, Martin Görner, Ralf-Ulrich Trappe, Christoph Röllig, Stefan Krause, Maher Hanoun, Olaf Hopfer, Gerhard Held, Sebastian Buske, Lars Fransecky, Sabine Kayser, Christoph Schliemann, Kerstin Schaefer-Eckart, Yousef Al-Fareh, Jörg Schubert, Thomas Geer, Martin Kaufmann, Arne Brecht, Dirk Niemann, Meinhard Kieser, Martin Bornhäuser, Uwe Platzbecker, Hubert Serve, Claudia D. Baldus, Carsten Müller-Tidow, Richard F. Schlenk

**Affiliations:** 1 Department of Internal Medicine V, Heidelberg University Hospital, Heidelberg, Germany; 2 Institute of Medical Biometry and Informatics, University of Heidelberg, Heidelberg, Germany; 3 NCT-Trial Center, National Center of Tumor Diseases, Heidelberg University Hospital and German Cancer Research Center, Heidelberg, Germany; 4 Department of Medicine IV, Aachen University Hospital, Aachen, Germany; 5 Department of Medicine II, Augsburg University Hospital, Augsburg, Germany; 6 Department of Hematology, Oncology and Palliative Medicine, Augusta Hospital Bochum, Bochum, Germany; 7 Department of Medicine and Polyclinic III, Bonn University Hospital, Bonn, Germany; 8 Department of Hematology, Oncology and Palliative Medicine, Community Hospital Bielefeld, Bielefeld, Germany; 9 Department of Medicine II, Prot. Diaconal Hospital Bremen, Bremen, Germany; 10 Department of Internal Medicine I, TU Dresden University Hospital, Dresden, Germany; 11 Department of Medicine V, Erlangen University Hospital, Erlangen, Germany; 12 Department of Hematology, Essen University Hospital, Essen, Germany; 13 Department of Medicine I, Hospital Frankfurt (Oder), Frankfurt (Oder), Germany; 14 Department of Internal Medicine I, Westpfalz Hospital Kaiserslautern, Kaiserslautern, Germany; 15 Department of Medicine II, Community Hospital Kiel, Kiel, Germany; 16 Department of Internal Medicine II, Schleswig-Holstein University Hospital Kiel, Kiel, Germany; 17 Department of Medicine I – Hematology and Cell Therapy, Leipzig University Hospital, Leipzig, Germany; 18 Department of Medicine A, Münster University Hospital, Münster, Germany; 19 Department of Internal Medicine V, North Hospital Nürnberg, Nürnberg, Germany; 20 Department of Hematology and Oncology, St. Josef Brothers’ Hospital Paderborn, Paderborn, Germany; 21 Department of Internal Medicine II, Elbland Hospital Riesa, Riesa, Germany; 22 Department of Medicine II, Diaconal Hospital Schwäbisch-Hall, Schwäbisch Hall, Germany; 23 Department of Hematology, Oncology and Palliative Medicine, Robert-Bosch Hospital Stuttgart, Stuttgart, Germany; 24 Department of Internal Medicine II, Helios Dr. Horst Schmidt Hospital Wiesbaden, Wiesbaden, Germany; 25 Department of Internal Medicine, Hematology, Oncology and Palliative Medicine, Prot. Monastery Hospital St. Jakob Koblenz, Koblenz, Germany; 26 Department of Hematology/Oncology, Johann Wolfgang Goethe University, Frankfurt, Germany

**Keywords:** gemtuzumab ozogamicin, glasdegib, acute myeloid leukemia, measurable residual disease

## Abstract

**Background:**

Overall survival remains poor in older patients with acute myeloid leukemia (AML) with less than 10% being alive after 5 years. In recent studies, a significant improvement in event-free, relapse-free and overall survival was shown by adding gemtuzumab ozogamicin (GO), a humanized antibody-drug conjugate directed against CD33, to intensive induction therapy once or in a sequential dosing schedule. Glasdegib, the small-molecule inhibitor of smoothened (SMO), also showed improved overall survival in patients not eligible for intensive chemotherapy when combined with low-dose cytarabine compared to low-dose cytarabine alone. These findings warrant further investigations in the phase III GnG trial.

**Methods/Design:**

This is a randomized phase III trial with measurable residual disease (MRD) after induction therapy and event-free survival (EFS) as primary endpoints. The two research questions are addressed in a 2 by 2 factorial design. Patients age 60 years and older are upfront randomized 1:1 in one of the two induction arms: GO administered to intensive induction therapy on days 1,4, and 7 versus GO administered once on day 1 (GO-147 versus GO-1), and double-blinded 1:1 in one of the subsequent treatment arms glasdegib vs. placebo as adjunct to consolidation therapy and as single-agent maintenance therapy for six months. Chemotherapy backbone for induction therapy consists of standard 7 + 3 schedule with cytarabine 200 mg/m^2^ continuously days 1 to 7, daunorubicin 60 mg/m^2^ days 1, 2, and 3 and high-dose cytarabine (1 g/m^2^, bi-daily, days 1, 2, and 3) for consolidation therapy. Addressing two primary endpoints, MRD-negativity after induction therapy and event-free survival (EFS), 252 evaluable patients are needed to reject each of the two null hypotheses at a two-sided significance level of 2.5% with a power of at least 85%.

**Ethics and dissemination:**

Ethical approval and approvals from the local and federal competent authorities were granted. Trial results will be reported via peer-reviewed journals and presented at conferences and scientific meetings.

**Trial status:**

Protocol version: 1st version 20.10.2020, no amendments yet. Study initiation on February 16, 2021. First patient was recruited on April 1st.

**Trial registration:**

ClinicalTrials.govNCT04093505; EudraCT 2019-003913-32. Registered on October 30, 2018.

**Supplementary Information:**

The online version contains supplementary material available at 10.1186/s13063-021-05703-w.

## Background

Acute myeloid leukemia (AML) is predominantly a disease of older patients for whom the prognosis is still poor [1, 2]. Intensive induction chemotherapy, usually consisting of an anthracycline and cytarabine, induces remission in about 50% of older fit patients, but most of these patients relapse and still succumb to their disease. Disease-related factors such as the genetic profile of the disease predict resistance to current standard therapy [3]. In line, the proportion of patients with a high-risk disease profile according to European LeukemiaNet (ELN)-2017 risk classification [4] increases with older age to roughly one-quarter of patients 70 years or older [5]. Combination of an anthracycline with cytarabine remains the standard of care of intensive induction therapy in patients considered medically fit [1, 2, 4] and the proportion of patients receiving intensive chemotherapy even in older patients is high with 80% to 90% in 60- to 70-year-old patients and 50% to 75% in patients aged between 70 and 75 years [5]. For patients who achieve a complete remission (CR) after induction chemotherapy, post-remission therapy is required to prevent relapse. However, despite intensive consolidation therapy, overall survival in older (≥ 60 years) patients remains poor with less than 10% being alive after 5 years [6]. Beyond age, genetic abnormalities constitute the most influential prognostic factors for survival [7, 8]. This is reflected in the current World Health Organization (WHO) classification of myeloid neoplasms and acute leukemia [9].

Gemtuzumab ozogamicin (GO) is a humanized immunoglobulin G4 antibody (hP67.6) directed against CD33 and conjugated to the DNA toxin calicheamicin via a hydrolyzable linker. GO/CD33 complexes are internalized into lysosomes, releasing calicheamicin and promoting single and double-strand breaks hereby inducing cellular death [10]. GO initially received accelerated FDA approval in 2000 for the treatment of patients aged ≥60 years with CD33 positive AML in first relapse [10]. Thereafter, a phase 3 study (S0106) was conducted by the Southwest Oncology Group (SWOG) in untreated de novo AML patients, comparing daunorubicin/cytarabine (DA) with 45 mg/m^2^ daunorubicin plus GO 6 mg/m^2^ on day 4 versus DA alone with 60 mg/m^2^ daunorubicin. The GO arm showed higher induction mortality (5.5% vs. 1.4%), without improving CR or relapse-free survival [11]. Based on these negative results, GO was withdrawn from the market in 2010. Meanwhile, results from five additional randomized studies with GO as adjunct to intensive induction therapy are available: Groupe Ouest Est d’Etude des Leuce´ mies aigue¨s et Autres Maladies du Sang (GOELAMS) AML2006IR [12], Medical Research Council (MRC) AML15 [13] and ALFA-0701 [14, 15], National Cancer Research Institute (NCRI) AML16 [16], and German-Austrian Acute Myeloid Leukemia Study Group (AMLSG) 09-09 [17]. ALFA-0701 randomized 278 patients aged 50 to 70 years with untreated de novo AML to either DA (60 mg/m^2^ daunorubicin) alone or to the same in combination with a fractionated GO induction schedule (3 mg/m^2^ on days 1, 4, and 7) [14]. Although CR with or without platelet recovery and early deaths were similar, patients in the GO arm had significantly improved median event-free (19.6 vs. 11.9 months; *P*=0.00018) and overall survival (OS) (34 vs. 19.2 months; *P*=0.046). A subgroup analysis revealed that the clinical benefit is mainly restricted to patients with favorable and intermediate-risk karyotype [14]. A meta-analysis of 3.325 patients (aged 18–84) from 5 randomized studies investigating GO as adjunct to induction chemotherapy in untreated AML concluded that the addition of GO improved OS in patients without adverse cytogenetics [18]. Rates of sinusoidal obstruction syndrome (SOS), a side effect associated with GO treatment, and 30- and 60-day mortality were lower with 3 mg/m^2^ vs. 6 mg/m^2^ GO [19]. Out of the five studies included in the meta-analysis, Castaigne et al. was the only one reporting on fractionated GO in a dosage of 3 mg/m^2^ on days 1, 4, and 7 (GO-147) [14, 18]. Interestingly, the addition of GO to induction therapy did not lead to an improved CR rate but a significantly higher rate of patients being negative for measurable residual disease (MRD-negative, 7% versus 39% in the standard and experimental arm, respectively) [20]. In addition, treatment with fractionated GO-147 was associated with a significant survival benefit in the large meta-analysis in comparison to patients that did not receive GO (OR:0.24, 99% CI 0.07–0.85), while this difference could not be shown for treatment with single-dose GO-1 (3 mg/m^2^) (OR: 1.0, 99% CI 0.78–1.3). Importantly, non-relapse mortality was not increased in patients treated with GO [14]. A major concern for patients receiving GO is the risk of SOS, especially among patients who received allogeneic hematopoietic cell transplantation (allo-HCT) within the preceding three months [21]. Revised dosing schedules significantly lowered rates of SOS to expected levels in patients being GO-naive [14, 22, 23]. Thus, the randomized comparison of GO-147 versus GO-1 as adjunct to intensive induction therapy appears as a logical consequence in terms of safety and efficacy [24].

The efficacy of GO during consolidation therapy was evaluated in 2 trials assessing GO on a randomized basis. In the MRC AML15 trial, a total of 948 patients were assigned to receive or not receive GO as adjunct to first consolidation therapy [13]. There were no differences in cumulative incidence of relapse (GO 46% vs.no GO 51% p=0.20) or OS (p=0.9) between the two groups. In the study from the Hemato-Oncologie voor Volwassenen Nederland (HOVON) group, older patients, who achieved CR after intensive induction therapy were randomized to either 3 cycles of GO (6 mg/m^2^ every 4 weeks) (*n*=113) or no postremission therapy (*n*=119) [25]. There were no significant differences regarding OS (*p*=0.52) and disease-free survival (*p*=0.40) between both groups. Thus, to date, no randomized data are available supporting the addition of GO in consolidation therapy [24, 26].

In AML, cytotoxic chemotherapy can reduce tumor bulk but is less effective at targeting tumor-initiating cells. The key challenge has been to identify the molecular mechanisms maintaining and sustaining tumor-initiating cell activity, self-renewal and survival. The Hedgehog (Hh) signaling is critical in terminal cell differentiation during embryogenesis and is believed to play a key role in the development of human malignancies when aberrantly activated. In AML aberrant activation of the Hh signaling pathway has been shown to be implicated in the maintenance of leukemia stem cell populations in several model systems [27]. Glasdegib is a selective, small-molecule inhibitor of smoothened (SMO), a membrane protein that regulates the Hh pathway. In vivo treatment of AML cells with glasdegib attenuated the leukemia-initiation potential in a serial transplantation mouse model [28]. Comprehensive gene set enrichment analysis revealed that glasdegib modulates self-renewal signatures and cell cycle progression [29]. Clinical data have supported these encouraging results. In a phase I study, a maximally tolerated dose of 400 mg daily was established and in a phase II study the recommended dose was 100 mg daily [30, 31]. In a randomized phase 2 study in older patients not fit for intensive chemotherapy, the addition of glasdegib 100 mg daily to low-dose cytarabine resulted in a significantly higher CR rate and OS as compared to low-dose cytarabine alone [32]. Interestingly, the beneficial effect of glasdegib on OS was not restricted to patients achieving a CR, as the observed beneficial effect on OS was larger than that seen on the CR-rate supporting the leukemic stem cell targeting effect of glasedib [32].

Based on the compelling preclinical data and the results of the phase-I and randomized phase-II studies, it appears reasonable and clinically feasible to combine standard intensive consolidation therapy with glasdegib. In this manuscript, we describe the rationale, design, and dosing details of the GnG study (clinicaltrials.gov identifier, NCT04093505; EudraCT No, 2019-003913-32), a phase III study to compare two schedules of GO as adjunct to intensive induction therapy and to compare intensive postremission therapy with or without glasdegib in a double-blinded manner in older patients with newly diagnosed AML.

Primary objectives of the study are (i) to assess the clinical efficacy of sequential or one-dose GO as adjunct to intensive induction therapy and (ii) to assess the clinical efficacy of glasdegib added to consolidation therapy and as a single agent for 6 months’ maintenance therapy in older patients with newly diagnosed AML.

## Methods

### Design

The GnG study is a randomized phase III trial with MRD after induction therapy and event-free survival (EFS) as primary endpoints. The two research questions are addressed in a 2 by 2 factorial design. Patients are upfront randomized to one of the two induction schedules (GO-147 versus GO-1) and to glasdegib or placebo (double blinded) added to consolidation therapy and as single agent for 6 months’ maintenance therapy in a 1:1 ratio. The trial is designed to gain evidence of the anti-leukemic activity of GO and glasdegib in older patients with newly diagnosed AML.

### Study setting and randomization

In Germany, patients with newly diagnosed AML are usually referred to academic Hospitals. In this study, patients will be recruited in 25 academic centers registered in the Study Alliance Leukemia (SAL) group. Participating centers are contacted by study monitors and the medical coordinator monthly to promote patient recruitment. Furthermore, after protocol amendments or upon relevant updates during the study, a newsletter will be sent to all participating centers. Conferences with the participating centers of the SAL network are done regularly to share information regarding therapy responses and complications seen within the study. Expecting at least 5 eligible patients per year and center, approximately 2 years are required to recruit the intended number of patients.

Each patient having signed informed consent and meeting all inclusion criteria is registered in the electronic case report form (eCRF). Via eCRF a unique patient ID (PAT-ID) is assigned. Following registration, eligible patients are upfront randomized 1:1 to induction chemotherapy containing either fractionated GO treatment (GO-147) or one single dose of GO (GO-1) and again 1:1 either to glasdegib or placebo (double-blinded) as adjunct to consolidation therapy and as single-agent for 6-months of maintenance therapy. Following these randomizations, 63 patients shall be allocated to each arm. Randomization is stratified by assumingly important prognostic factors age (≤70 years vs. > 70 years) and Eastern Cooperative Oncology Group (ECOG) performance status (PS) (ECOG PS = 0 vs. ECOG PS > 0). Block randomization with varying block lengths is used and performed using the web tool www.randomizer.at, by which randomization for double-blind clinical trials can easily be handled. Patients withdrawn from the trial retain their identification codes. Patients have to provide written informed consent before any protocol-specific procedures are performed. Applicable regulatory requirements, Good Clinical Practice, and ethical principles from the Declaration of Helsinki are adhered to during the study.

### Post-randomization events

If MRD-negativity cannot be measured, the outcome will be imputed. Any-cause death before MRD measurement, will be regarded as MRD-positive. Dropouts (including lost-to-follow-up) are considered as censoring events. The dropout rate for the assessment of the short-term endpoint is assumed to be 3%. For the long-term endpoint EFS, a dropout rate of 5% is expected 2 years after randomization. Dropout times are assumed to be exponentially distributed.

### Withdrawal of patients

A patient must be withdrawn from the trial (i) at any time at the patient’s own request, (ii) after induction therapy if the patient fails to obtain CR/CRi, (iii) at any time if unacceptable toxicity necessitating cessation of treatment is observed, (iv) at any time if there are changes in the medical status of the patient that compromise the patient’s safety or if the investigator considers that the withdrawal is in the patient’s best interest, (v) in case of pregnancy, (vi) at any time if insufficient protocol compliance from the patient is observed, (vii) if the patient is lost to follow-up. Patients who meet criteria i) to vi) and agree with the continuation of follow-up will be followed according to the protocol (supplementary Table 5.).

Unresolved AEs are followed in such cases; however, if the patient withdraws from the trial and also from consent for disclosure of future information (e.g., follow-up visits), further data collection is prohibited.

### Treatments and study procedures

#### Induction therapy

Patients receive one cycle of backbone induction therapy with standard 7 + 3 regimen: cytarabine 200 mg/m^2^ administered via continuous intravenous (IV) infusion for a total of 7 days and daunorubicin 60 mg/m^2^ days 1, 2, and 3. Patients are randomized to receive in addition GO 3 mg/m^2^ IV over 1 h (Mylotarg®), either on days 1,4, and 7 or only once on day 1 (GO-147 versus GO-1). Dose modification in case of CTC grade ≤2 toxicity is allowed in the GO-147 schedule to enable continued administration of GO on day 4 and day 7, respectively. In the case of grade 3 toxicity on day 1 and/or 4, patients will receive GO on days 4 and 7, respectively, if the CTC grade has improved to grade < 3 toxicity prior to infusion. In the case of CTC grade 4 toxicity, GO is discontinued. Likewise, patients who develop anaphylaxis, pulmonary edema, acute respiratory distress syndrome, or SOS after the first administration are not allowed to receive further doses of GO. On days 15 and 28 (window day 28 to day 42), a bone marrow aspirate specimen is collected for local and central assessment. If this bone marrow specimen is not evaluable for assessment of response, the bone marrow aspiration has to be repeated upon count recovery or day 42 whichever occurs first. In case of bone marrow blast count > 10% on day 15, or no CR or CR with incomplete neutrophil or platelet recovery (CRi) after induction therapy, one cycle of HAM (high dose cytarabine and mitoxantrone) as salvage therapy is allowed within the protocol.

#### Consolidation and maintenance therapy

During the consolidation phase, patients receive up to two cycles of cytarabine (1.0 g/m^2^) administered by IV infusion every 12 h on days 1, 2, and 3 [33]. Study drug (glasdegib 100 mg or placebo) is orally administered with approximately 8 ounces (240 mL) of water in the morning, at the same time each day from cycle day 1 to 28. Cycle 2 of consolidation chemotherapy is scheduled to start immediately after the end of cycle 1 or within the next two weeks if blood count recovery is delayed. In case of hematologic toxicity, a dose reduction or delay of glasdegib is not required. Remission status assessments take place after each consolidation therapy cycle. Patients may undergo allo-HCT after induction or after any of the consolidation therapy cycles.

**Fig. 1:**
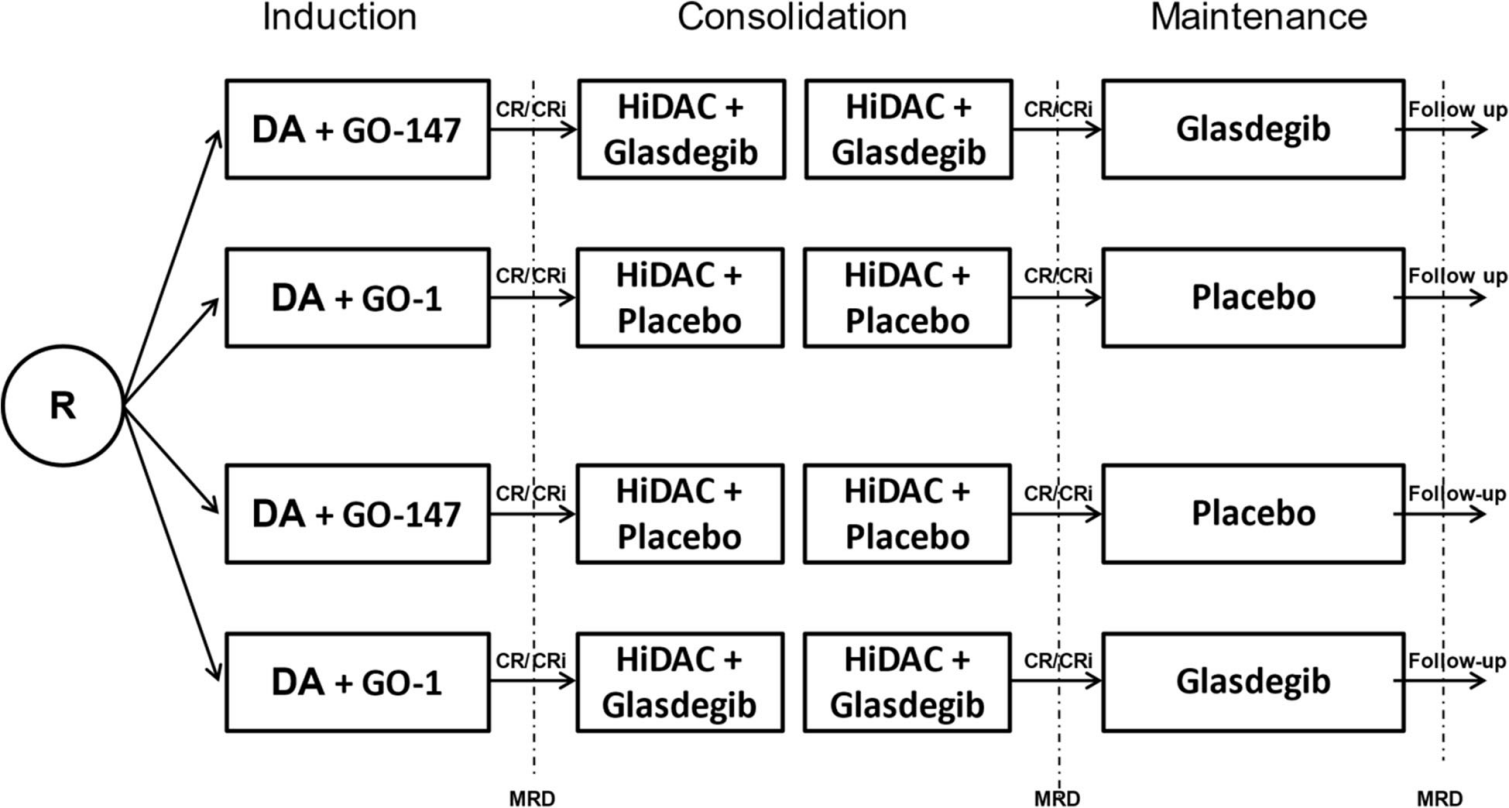
Overall treatment schedule GnG-Study. Abbreviations: DA, daunorubicin; low-dose cytarabine; GO, gemtuzumab ozogamicin; HiDAC, high-dose cytarabine (1 g/m^2^); MRD, measurable residual disease; CR, complete remission; CRi, CR with incomplete hematological recovery. In case of bone marrow blast count > 10% or no CR/CRi after on day 15 after induction therapy one cycle of HAM (high-dose cytarabine and mitoxantrone) is allowed. Maintenance is intended in all patients in CR/CRi irrespective of completion of consolidation therapy

During maintenance therapy, the dose of the study drug is the same as during consolidation therapy (glasdegib 100 mg). Maintenance therapy with glasdegib or placebo begins after the end of the 2nd consolidation therapy cycle (includes recovery period of up to 14 days, if applicable) and after assessment of remission status or 180 days after allogeneic HCT. Patients receive up to 6 cycles of 28 days each (168 days in total) within the maintenance schedule. Remission status assessments take place every three months for two years after beginning maintenance therapy. The overall treatment schedule is summarized in Fig. 1.

Glasdegib and placebo are interrupted in patients experiencing adverse events of grade 3 or 4. Appropriate follow-up assessments are performed until adequate recovery from toxicity. In patients recovering within 21 days from dose interruption, glasdegib/placebo may be resumed. If hematological recovery parameters are not met after 21 days of dose interruption, permanent discontinuation of treatment with glasdegib/placebo is advised. Criteria for dose interruption and dose reductions in cases of non-hematological toxicities including applicable doses in milligrams are summarized in Tables 1 and 2.

**Table 1:** Glasdegib interruptions in case of toxicities

Toxicity causing glasdegib interruption	Resumption within the first 21 days when:
Any toxicity grade ≥3 according to CTCAE criteria potentially attributable to glasdegib regardless of when it occurs in the cycle.	Toxicity returns to patient’s baseline/ toxicity resolved (non-hematological toxicity recovers to grade ≤1)
ANC < 0.1G/l and /or platelets <10G/l regardless of when it occurs in the cycle	ANC ≥0.1G/l and platelet count ≥10G/l and re-treatment can occur safely as per the investigator’s judgment
No resolution of above toxicities after 21 days	Discontinue medication permanently

Glasdegib doses omitted for toxicity are not replaced within that cycle (e.g., cycles are not to be prolonged beyond 28 days in order to make up for any missed glasdegib doses during that cycle). Toxicity is graded according to CTCAE criteria. Once the Glasdegib dose has been reduced, all subsequent cycles should be administered at that dose level, unless further dose reduction is required. Dose re-escalation is not allowed. Abbreviations: *CTCAE* Common Terminology Criteria for Adverse Events, *ANC* absolute neutrophil count.

**Table 2:** Glasdegib dose reduction in case of non-hematological toxicities

Toxicity	Glasdegib dosage modification
Non-hematologic toxicities grade ≥3 according to CTCAE criteria (excluding QTc prolongation, muscle spasms, and myalgias). First episode Second episode Third episode	Interrupt medication until toxicity recovers to grade ≤1, then: Dose level decrease 1 (DLD1): 75 mg DLD2: 50 mg Discontinue medication permanently
Renal toxicity, where serum creatinine or BUN are ≥2 × ULN or serum bicarbonate level is < 20 mmol/L. First episode Second episode Third episode	Interrupt medication until toxicity recovers tograde ≤1 then: DLD1 DLD2 Discontinue medication permanently
Electrocardiogram QT corrected (QTc) prolongation grade 1.	Continue at the same level.
QTc prolongation grade 2 and 3.	Interrupt and resume when QTc returns to ≤470 ms: - Within 7 days, dosing as before - Within 14 days, DLD1 Discontinue medication permanently, in case of no return to ≤470 ms after 14 days,
QTc prolongation grade 4 or repetitive grade 3 or grade 2 after DLD1.	Discontinue medication permanently

Toxicity is graded according to CTCAE criteria. Once the Glasdegib dose has been reduced, all subsequent cycles should be administered at that dose level, unless further dose reduction is required. Dose re-escalation is not allowed. Nausea, vomiting, or diarrhea must persist until next therapy cycle at grade ≥3 to require dose modification

Abbreviations: *CTCAE* Common Terminology Criteria for Adverse Events, *QTc* QT corrected, *DLD1* dose level decrease 1: 75 mg, *DLD2* dose level decrease 2: 50 mg, *ULN* upper limit normal

#### Long-term follow-up

The period of observation under therapy ends with the last visit of the sixth cycle of maintenance therapy. After the end of treatment visit, patients are routinely followed-up according to standard of care. Follow-up is intended until the last patient alive has been observed for at least 2 years (study treatment including subsequent follow-up). Assuming 2 years of linear recruitment, total observation of the first patient may last up to 4 years and a median follow-up of 3 years at end of study is expected.

Event-free survival and OS observational follow-up are recorded until the end of the study. After achieving an observation period of 2 years counted from day 1, the follow-up may be performed by contacting the treating physician instead of in house-visits.

#### Additional study procedures during induction, consolidation, and continuation phases

Patients undergo efficacy and safety assessments, including monitoring of MRD, bone marrow specimen collection, blood and urine sampling, and patient-reported outcomes before receiving study drug and at specified time points throughout the study (supplementary Tables 1 to 4).

### Participants

#### Inclusion criteria

Inclusion criteria are outlined in Table 3. Key inclusion criteria are newly diagnosed AML according to the 2016 WHO classification, no prior chemotherapy for leukemia except hydroxyurea for up to 7 days to control hyperleukocytosis, age 60 years and older, and ECOG PS between 0 and 2.

**Table 3:** Inclusion and exclusion criteria

Category	Inclusion	Exclusion
Population characteristics	- Patients with newly diagnosed acute myeloid leukemia according to the 2016 WHO classification. - Genetic and immunophenotypic assessment in one of the central laboratories. - Age ≥ 60 years, no upper age limit. - ECOG performance status ≤2. - Effective contraception method.	- AML with PML-RARA or BCR-ABL1. - Patients with known active CNS leukemia. - Pregnancy and lactation. - Known or suspected active alcohol or drug abuse. - Known positivity for HIV, active HBV, HCV, or hepatitis A infection. - Severe neurologic or psychiatric disorder interfering with ability of giving informed consent.
Prior therapies	- No prior chemotherapy for leukemia except hydroxyurea to control hyperleukocytosis (≤7 days).	- Prior treatment with a smoothened inhibitor (SMOi) and/or hypomethylating agent.
Comorbidities		- Inadequate renal function. - Inadequate liver function. - Known liver cirrhosis. - History of sinusoidal. Obstruction syndrome. - Uncontrolled hypertension. - Severe obstructive restrictive. ventilation disorder. - Myocardial infarction. - Congenital long QT syndrome. - Torsades de pointes. - Arrhythmias (including sustained ventricular tachyarrhythmia). - Right or left bundle branch block and bifascicular block. - Unstable angina. - Coronary/peripheral artery bypass graft. - Symptomatic congestive heart failure (NYHA III/IV). - Cerebrovascular accident. - Transient ischemic attack. - Symptomatic pulmonary. embolism. - Bradycardia defined as < 50 bpms. - QTc interval > 470 msec. - Uncontrolled infection. - Evidence or history of severe non-leukemia associated bleeding diathesis or coagulopathy. - Patients with a “currently active” second malignancy other than non-melanoma skin cancer.
Others	- Signed written informed consent. - Ability of the patient to understand character and consequences of the clinical trial.	- No consent for biobanking. - History of hypersensitivity to the investigational medicinal product or to any drug with a similar chemical structure. - Participation in a clinical study involving an investigational drug.

Abbreviations: *AML* acute myeloid leukemia, *CNS* central nervous system, *ECOG* Eastern Cooperative Oncology Group, *NYHA* New York Heart Association

#### Exclusion criteria

Exclusion criteria are summarized in Table 3. Main exclusion criteria are diagnosis of acute promyelocytic leukemia (APL) with translocation t(15;17)(q22;q12) or *BCR-ABL*-positive AML. Other exclusion criteria are known active CNS leukemia, HIV, viral hepatitis, prior treatment with a smoothened inhibitor (SMOi) and/or hypomethylating agent, as well as known liver cirrhosis or history of SOS.

### Efficacy

The GnG trial has two efficacy endpoints. The first is MRD-negativity after sequential or single-dose GO in combination with intensive induction therapy. MRD-negativity is defined as the absence of leukemic cells at the end of the induction therapy assessed by flow cytometry with a sensitivity of 10^-4-^10^-5^. If MRD-negativity cannot be measured, or if patients drop out of the study before MRD measurements, missing values will be replace using multiple imputation. Patients who die from any cause before MRD measurement will be regarded as MRD-positive. The second endpoint is EFS after two years; EFS is defined as the time from randomization until one of the following events, whichever occurs first: a) failure to obtain CR or CR with incomplete neutrophil or platelet recovery (CRi) after induction therapy, b) relapse from CR/CRi or c) death from any cause. Patients without an event are censored at last follow-up. Refractory disease or treatment failure is defined as failure to achieve CR or CRi, presence of Auer rods, or appearance of new or worsening extramedullary disease after induction therapy. Relapse after CR or CRi is characterized by ≥5% blast cells in the bone marrow aspirate and/or biopsy not attributable to any other cause, the reappearance of leukemic blasts in the peripheral blood, appearance of extramedullary leukemia, or presence of Auer rods. Platelet (≥100 G/l) and neutrophil (≥1.0 G/l) counts for the assessments of CR and CRi are assessed according to standard criteria [4].

Secondary survival endpoints are OS (defined as time from randomization until death from any cause) and relapse-free survival (RFS) (measured from first CR/CRi to time of recurrence of the disease or death from any cause, whichever occurs first). Patients without an event are censored at the last date of follow-up. Further secondary endpoints are response (CR/CRi) after induction therapy, patient-reported outcomes (PROs) and pharmacoeconomics. PROs include assessments of a) health-related quality of life (QoL), calculated as the EORTC QLQ-C30 Summary Score [34], b) the quality of sleep or sleep disorders, calculated with the “Sleep Quality Index” from the PSQI according to the corresponding scoring guidelines [35], and c) anxiety and depression, calculated from the PHQ-4 according to the corresponding scoring manual [36]; pharmacoeconomics with health care resource utilization is assessed by self-administered resource utilization questionnaire and the SF-36 [37] [38] questionnaires for health economic analyses with patient-reported information on personal traits and experiences are collected at baseline.

### Safety assessments

All adverse events (AEs) that occur after the clinical screening visit (or as soon as the medical history of the patient has been examined) are documented. The period of observation ends with the last study visit. All patients who have AEs, whether considered associated with the use of the investigational medical products or not, are monitored for outcome determination. All AEs are coded using the latest version of the Medical Dictionary for Regulatory Activities and assigned grades based on National Cancer Institute Common Terminology Criteria for Adverse Events, version 5.00. The Data Monitoring Committee (DMC) reviews all data relevant to safety. The DMC, which is composed of three independent experts meets regularly and provides the sponsor with recommendations regarding trial modification, continuation, or termination.

### Auditing

Audits are planned to be performed based on the regular risk-based evaluation. Regulatory authorities and auditors authorized by the sponsor may request access to all source documents, the CRF, and other trial documentation. Investigators are contractually bound to enable direct access to these documents and to support audit activities.

### Protocol amendments

Decisions regarding protocol amendments will be taken by the study core team encompassing the coordinating investigator, trial coordinator, trial statistician, medical coordinator, and data management. Meetings for reviewing all available findings and information are scheduled every 2 weeks.

### Data collection and handling

All data required as per the study protocol, including clinical and laboratory data, are documented by the investigator or an authorized member of the study team in the medical record of the patient and in the eCRF. Access to the eCRF is password protected and an audit trail is in place. Any entries are tracked and locked to prevent further editing. The investigator at the clinical site is responsible for ensuring that all sections of the eCRF are completed correctly. Entries are checked for plausibility and consistency via eCRF-inherent edit checks and visually by the monitors where necessary. Implausibility and missing entries are queried and to be clarified with the responsible investigator. All relevant documents and data collected within the study will be archived for at least 10 years after the termination of the study.

### Study Monitoring

Study monitoring is done by the Heidelberg Clinical Studies Coordination Center (KKS). A total of 130 monitoring visits to 25 study sites (5 per site on average) are planned over a study period of 48 months. The first monitoring visit at each study center is scheduled to occur at the end of the second patient's induction therapy. Further monitoring visits at each study site will depend on the (i) recruitment of study participants, (ii) the monitor's assessment of the trial site’s compliance with applicable stipulations (e.g., number and severity of protocol deviations or deficiencies detected during study visits), (iii) the deficiencies detected in the Central Data Review, and (iv) the assessment of the coordinating team. The monitoring is carried out according to a monitoring manual giving comprehensive guidance on monitoring activities (SDV rules, corrective and preventive actions, documentation of protocol violations, escalation of findings etc.).

### Ancillary and post trial care

The treatment period (EOT) ends after the last visit of the sixth cycle of maintenance therapy or may end prematurely for various reasons. After EOT patients are routinely followed up and treated as per the standard of care at the discretion of the treating physician. The period of observation ends for all patients when the last patient being included and alive has been followed for at least 730 days (2 years) counted from this patient’s day 1 of study treatment.

### Ethical and legal aspects

Before the start of the trial, the trial protocol, informed consent document, and any other appropriate documents were submitted to the independent Ethics Committee (EC) as well as to the competent federal authority (BfArM). A written favorable vote of the EC and an approval by the competent higher federal authority are a prerequisite for initiation of the clinical trial. All the procedures set out in this trial protocol are designed to ensure that all persons involved in the trial abide by Good Clinical Practice (GCP) and the ethical principles described in the current version of the Declaration of Helsinki. The trial is carried out in keeping with local legal and regulatory requirements. Before being admitted to the clinical trial, all patients must consent in written form to participate after the nature, scope, and possible consequences of the clinical trial have been understood by the patient.

All planned substantial changes to the study (protocol amendments) are to be submitted in writing to the EC and the competent federal authority requesting their approval. . Records of relevant communication with the EC and the regulatory authorities are kept by the coordinating investigator.

### Access to data and dissemination policy

After the publication of the complete trial, access to selected raw data is intended. This must be done in accordance with the European data protection act and informed consent given by the patients.

The results from this trial will be presented at national (e.g., annual meeting of the German Society of Hematology/Oncology); meetings of the Competence Net “Acute and Chronic Leukemias” and international meetings (e.g., meetings of the European Leukemia-Net; annual congresses of the European Hematology Association, the American Society of Hematology and the American Society of Clinical Oncology). The full results will be published in high-impact peer-reviewed medical journals.

### Sample size calculation and statistics

Addressing two primary endpoints, MRD-negativity after induction therapy and EFS, 252 evaluable patients are needed to reject each of the two null hypotheses at a two-sided significance level of 2.5% with a power of at least 85%.

The first primary endpoint evaluation involves the comparison of rates of MRD-negativity assessed by flow-cytometry after induction therapy between GO-147 and GO-1. Assuming a rate of MRD-negativity of 45% for GO-147 and 20% for GO-1, as well as a 3% dropout rate, a total number of 252 evaluable patients are needed to reject the null hypothesis of no difference regarding the MRD-negativity rate for patients receiving GO-147 as compared to patients receiving GO-1 during induction therapy at a two-sided significance level of 2.5% with a power of at least 85% using a chi-squared test.

The second primary endpoint evaluation involves a two-group comparison of EFS between the experimental arm of glasdegib as well as the control arm of placebo both as adjunct to standard consolidation therapy. Assuming a 2-year EFS of 38.5% for the experimental arm and a 2-year EFS of 21% for the control arm (resulting in a hazard ratio of HR=0.612), as well as an exponentially distributed dropout rate of 5% at 2 years, a total number of 224 evaluable patients (based on a number of *d*=178 required events) are needed to reject the null hypothesis assuming no difference regarding EFS for patients receiving glasdegib as compared to patients receiving placebo at a two-sided significance level of 2.5% with a power of at least 85% using a log-rank test, assuming an accrual time of 24 months, as well as a follow-up time of 24 months. This leads to a total sample size of N=max(252, 224)=252 patients to be enrolled for the whole trial to ensure a power of at least 85% for both primary endpoints.

The MRD-negativity after induction therapy is analyzed using a generalized linear mixed model and EFS with a Cox regression frailty model. Both models are adjusted for the following fixed factors: treatment (MRD-negativity: GO-1 vs. GO-147 and EFS: glasdegib vs. placebo), age, sex, and ECOG PS, as well as for the random factor “recruiting center”. The primary analysis is based on the full analysis set including all randomized patients. Adjustment for multiple testing is done using the Bonferroni-Holm procedure in order to control the family-wise error rate at a two-sided significance level of 5% in the strong sense. Missing values for the short-term primary endpoint MRD-negativity are replaced using multiple imputation by using the fully conditional specification method [39]. Odds and hazard ratios are reported alongside with two-sided 97.5% and 95% confidence intervals, and a possible center effect is assessed by calculating the intra-class correlation coefficient and by presenting the results stratified for the center. A sensitivity analysis of the long-term primary endpoint additionally includes the interaction between maintenance therapy and induction therapy. Statistical analysis is performed using SAS v9.4 or higher.

## Discussion

We designed a randomized phase-III study to compare two schedules of GO as adjunct to intensive induction therapy and to compare intensive postremission therapy with or without glasdegib (GnG-study) in a double-blinded manner. This study intends to answer two research questions: first, whether fractionated GO administered on days 1, 4, and 7 outperforms a single dose of GO on day 1 during induction therapy with the endpoint MRD status after induction therapy, and second, whether glasdegib as adjunct to consolidation therapy and as single-agent maintenance therapy for six months improves EFS.

According to the meta-analysis of Hills et al. [18], the addition of GO to induction chemotherapy significantly reduced the risk of relapse. The clinically most relevant effect was seen in the ALFA-0701 trial (risk of relapse; HR, 0.55), which administered GO on days 1, 4, and 7, compared to GO on day 1 in the MRC trials (risk of relapse; HR, 0.82). This reduction led to an improvement in survival after achieving CR and OS [18]. However, already GO-1 as adjunct to intensive induction therapy has been shown to reduce significantly the MRD level in AML with mutated *NPM1* after induction therapy [37]. Still, it is unclear which GO regimen is more effective in achieving MRD-negativity. In addition, it is of high interest whether MRD status after induction therapy can serve as a surrogate outcome for survival.

MRD-negativity assessed by real time quantitative polymerase chain reaction (RT-qPCR) in patients with AML achieving CR is known to be associated with a lower relapse risk. It can be considered a broad predictive biomarker useful to guide the patient’s postremission management [40–44]. Thus, the ELN consensus recommends molecular MRD assessments for *NPM1* mutations, *RUNX1*-*RUNX1T1*, *CBFB*-*MYH11*, and *PML*-*RARA* fusion transcripts at diagnosis, after two cycles of induction/consolidation therapy, and every 3 months, for 24 months after the end of treatment [4]. However, MRD assessment by RT-qPCR can only be applied to AML patients with suitable molecular aberrations.

In the NCRI AML16 trial, flow cytometry was used for the detection of MRD in 186 AML patients in remission. The authors found no significant improvement in the quality of remission regarding MRD-negativity between patients receiving GO vs. control [16]. However, the addition of GO to induction therapy in a fractionated schedule in the ALFA-0701 trial led to a higher rate of patients being negative for MRD [20]. A recent meta-analysis, including 19 studies, concluded that, overall, pre-transplant MRD-positivity was associated with worse leukemia-free survival (HR, 2.76 [1.90–4.00]), OS (HR, 2.36 [1.73–3.22]), and cumulative incidence of relapse (HR, 3.65 [2.53–5.27]). However, the significant heterogeneity among studies using flow-based methods was observed, most likely due to site-specific methodological differences [45].

The multicenter AML02 study, which enrolled pediatric patients, showed that MRD assessed by flow cytometry after induction therapy was a better predictor of EFS, relapse rate, and RFS than the morphological assessment of treatment response [46].

In line with these findings, our first research question is whether GO applied in a fractioned manner increases the probability of MRD-negativity after induction therapy. Furthermore, we are aiming to evaluate if there is a correlation between MRD-negativity, as assessed by flow cytometry and relapse risk and survival in AML patients.

A correlation between MRD-positivity and relapse risk suggests that relapse is initiated by residual leukemia stem cells (LSC), which have shown to be resistant to conventional cytotoxic chemotherapy. In preclinical studies, glasdegib induced rapid and complete tumor regression as a single-agent or in combination with chemotherapy and reduced the expression of key leukemia stem-cell regulators hereby decreasing the leukemia stem-cell populations in patient-derived AML cells [27]. Thus, in our trial, we sought to investigate the combination of initial leukemia elimination by conventional chemotherapy and GO during the induction therapy phase and targeting of residual leukemic stem cells during consolidation and maintenance therapy with glasdegib. Efficacy of the addition of glasdegib is assessed by EFS as primary and OS as a secondary endpoint. EFS has been accepted as primary endpoint for the approval of GO in first-line therapy in AML by the FDA and EMA [47]. EFS compared to OS provides the advantage to be measurable earlier and to be directly linked to the treatment under investigation [39–50]. In contrast to overall survival, where death is the only event of interest, EFS also includes failure to obtain complete remission and relapse from complete remission. Thus, we assume that EFS as one primary endpoint will be able to better discriminate the potential contributions of the different therapeutic components (induction, consolidation, maintenance) to the overall response.

The strength of the current study is also one of its weaknesses. The 2 by 2 factorial design allows us to compare four therapy regimens. Based on known mechanisms of actions and the timely distinct use, GO in induction and glasdegib in postremission, we estimate that there will be no biometrical interaction between the investigational medical products in the trial design. Results from the meta-analysis on GO indicate that the clinical impact of GO given during induction therapy is independent of variations in consolidation therapy [18]. However, in the unlikely case of an interaction between therapies, sample size may not be sufficient to properly evaluate this interaction.

Submission to the independent Ethics Committee and the competent federal authority was completed in July 2020, and final approval was completed in November 2020. The first patient was recruited on the first of April 2021.

## Supplementary Information

**Additional file 1: Supplementary Tables. Table S1.** Detailed Description of Study Visits (Day by Day) induction therapy. **Table S2.** Detailed Description of Study Visits (Day by Day) salvage therapy. **Table S3.** Detailed Description of Study Visits (Day by Day) consolidation therapy. **Table S4.** Detailed Description of Study Visits (Day by Day) maintenance therapy. **Table S5.** Detailed Description of Study Visits (Day by Day) follow-up

**Additional file 2.** SPIRIT Checklist for Trials

## Abbreviations

CTCAE: Common Terminology Criteria for Adverse Events
ANC: Absolute neutrophil count
AML: Acute myeloid leukemia
ELN: European LeukemiaNet
CR: Complete remission
WHO: World Health Organization
GO: Gemtuzumab ozogamicin
SWOG: Southwest Oncology Group
DA: Daunorubicin/cytarabine
GOELAMS: Groupe Ouest Est d’Etude des Leuce´ mies aigue¨s et Autres Maladies du Sang
MRC: Medical Research Council
NCRI: National Cancer Research Institute
AMLSG: German-Austrian Acute Myeloid Leukemia Study Group
MRD: Measurable residual disease
allo-HCT: Allogeneic hematopoietic cell transplantation
HOVON: Hemato-Oncologie voor Volwassenen Nederland
Hh: Hedgehog
SMO: Small-molecule inhibitor of smoothened
OS: Overall survival
EFS: Event-free survival
ECOG: Eastern Cooperative Oncology Group
PS: Performance status
IV: Intravenous
CRi: Complete remission with incomplete neutrophil or platelet recovery
APL: Acute promyelocytic leukemia
SMOi: Smoothened inhibitor
SOS: Sinusoidal obstruction syndrome
RFS: Relapse-free survival
PROs: Patient-reported outcomes
QoL: Health-related quality of life
AE: Adverse event
DMC: Data Monitoring Committee
RT-qPCR: Real-time quantitative polymerase chain reaction
LSC: Leukemia stem cells
