# Supplementary material for: Rationale and design of the 2 by 2 factorial design GnG-trial: a randomized phase-III study to compare two schedules of gemtuzumab ozogamicin as adjunct to intensive induction therapy and to compare double-blinded intensive postremission therapy with or without glasdegib in older patients with newly diagnosed AML
Source: Trials. 2021 Nov 3;22:765. doi: 10.1186/s13063-021-05703-w (PMC8564967; doi:10.1186/s13063-021-05703-w)
Supplement: Supplementary file 1 — Additional file 1: Supplementary Tables. Table S1. Detailed Description of Study Visits (Day by Day) induction therapy. Table S2. Detailed Description of Study Visits (Day by Day) salvage therapy. Table S3. Detailed Description of Study Visits (Day by Day) consolidation therapy. Table S4. Detailed Description of Study Visits (Day by Day) maintenance therapy. Table S5. Detailed Description of Study Visits (Day by Day) follow-up [file 13063_2021_5703_MOESM1_ESM.docx]

**Supplementary Tables**

## Table 1. Detailed Description of Study Visits (Day by Day) induction therapy

|  | | **Induction therapy^1^ (IT), one induction cycle** | | | | | | | | | | | | |
| --- | --- | --- | --- | --- | --- | --- | --- | --- | --- | --- | --- | --- | --- | --- |
| **Phase** | **Baseline** | **IT therapy** | | | | | | | **IT recovery, duration of 3-5 weeks** | | | | | **End of cycle** |
| **DAY of Cycle [optional]** | -14-0 | 1 | 2 | 3 | 4 | 5 | 6 | 7 | 8 | 15* | 22* | [29*] | [36*] | EOC^2^ |
| Clinical assessments |  |  |  |  |  |  |  |  |  |  |  |  |  |  |
| Signs/symptoms | X |  |  |  |  |  |  |  |  |  |  |  |  | x |
| Vital signs | X^Height^ | x |  |  | x |  |  | x | x^W^ | x^W^ | x^W^ | [x^W^] | [x^W^] | x |
| Physical examination | X | x^o^ |  |  | x |  |  | x | x^W^ | x^W^ | x^W^ | [x^W^] | [x^W^] | x |
| ECG | X | X^o^ |  |  |  |  |  |  |  |  |  |  |  |  |
| Extramedullary involvement | X |  |  |  |  |  |  |  |  |  |  |  |  | X |
| ECOG PS | X | X^o^ |  |  | x |  |  | X | x^W^ | x^W^ | x^W^ | [x^W^] | [x^W^] | X |
| Laboratory assessments |  |  |  |  |  |  |  |  |  |  |  |  |  |  |
| Hematology | X | x^SL^ |  |  | x^SL^ |  |  | x^SL^ | x^SL,W^ | x^SL,W^ | x^SL,W^ | [x^SL,W^] | [x^SL,W^] | x |
| Basic blood chemistry | X | x^SL^ |  |  | x^SL^ |  |  | x^SL^ | x^SL,W^ | x^SL,W^ | x^SL,W^ | [x^SL,W^] | [x^SL,W^] | x |
| Extended blood chemistry  & coagulation | X | X^SL^ |  |  | X^SL^ |  |  | X^SL^ | x^SL,W^ | x^SL,W^ | x^SL,W^ | [x^SL,W^] | [x^SL,W^] | X |
| Local disease assessment | X |  |  |  |  |  |  |  |  |  |  |  |  | X |
| Central laboratory assessments |  |  |  |  |  |  |  |  |  |  |  |  |  |  |
| Sample collection (BM, PB) | X |  |  |  |  |  |  |  |  | X^3^ |  |  |  | x |
| MRD & Disease status | X |  |  |  |  |  |  |  |  |  |  |  |  | X |
| PROs and Health economics |  |  |  |  |  |  |  |  |  |  |  |  |  |  |
| Patient Reported Outcomes | x |  |  |  |  |  |  |  |  |  |  |  |  | X |
| Resource utilization questionnaire |  |  |  |  |  |  |  |  |  |  |  |  |  | X |
| Treatment |  |  |  |  |  |  |  |  |  |  |  |  |  |  |
| GO-147 (experimental arm) |  | x |  |  | x |  |  | x |  |  |  |  |  |  |
| GO-1 (control arm) |  | x |  |  |  |  |  |  |  |  |  |  |  |  |
| SOC: Cytarabine |  | X | X | X | X | X | X | X |  |  |  |  |  |  |
| SOC: Daunorubicin |  | X | X | X |  |  |  |  |  |  |  |  |  |  |
| Safety |  |  |  |  |  |  |  |  |  |  |  |  |  |  |
| Concomitant medications & treatment | X | x | x | x | x | x | x | x | x^W^ | x^W^ | x^W^ | [x^W^] | [x^W^] | x |
| AE assessment |  | x | x | x | x | x | x | x | x^W^ | x^W^ | x^W^ | [x^W^] | [x^W^] | x |
| Pregnancy test  (WOCBP only) | X | x^o^ |  |  |  |  |  |  |  |  |  |  |  |  |
| Screening and Baseline |  |  |  |  |  |  |  |  |  |  |  |  |  |  |
| Informed consent | X | **Footnotes:**  ^1^ conditional salvage therapy cycle (see next page) not considered  ^2^ includes treatment-free recovery period of 3-5 weeks, may be omitted in case   of ITSC.  * approximate number of day for assessments in weekly intervals   (i.e. day given as calculated)  Height=at baseline incl. height in cm  O=to be omitted if done within preceding 48h  SL=Safety lab, values not captured in eCRF  W=to be done in weekly intervals (preferably same day per week)  ^3^ optional | | | | | | | | | | | | |
| Demographics | X |  |  |  |  |  |  |  |  |  |  |  |  |  |
| Medical/oncologic history | X |  |  |  |  |  |  |  |  |  |  |  |  |  |
| Genetic assessment  (central lab) | x |  |  |  |  |  |  |  |  |  |  |  |  |  |
| Cytogenetics | x |  |  |  |  |  |  |  |  |  |  |  |  |  |
| ECHO | X |  |  |  |  |  |  |  |  |  |  |  |  |  |
| Abdominal ultrasound | x |  |  |  |  |  |  |  |  |  |  |  |  |  |
| Urinalysis | X |  |  |  |  |  |  |  |  |  |  |  |  |  |
| Virus diagnostics | X |  |  |  |  |  |  |  |  |  |  |  |  |  |
| Enrollment & Randomization | X |  |  |  |  |  |  |  |  |  |  |  |  |  |

## Table 2. Detailed Description of Study Visits (Day by Day) salvage therapy

|  | | **Induction therapy, one conditional^1^ salvage therapy cycle (IT-SC) potentially added after the induction cycle** | | | | | | | | | | | | |
| --- | --- | --- | --- | --- | --- | --- | --- | --- | --- | --- | --- | --- | --- | --- |
| **Phase** | **IT** | **IT-SC therapy** | | | | | | | **IT-SC recovery** | | | | | **End of cycle** |
| **DAY of Cycle [optional]** | 15/EOC | 1 | 2 | 3 | 4 | 5 | 6 | 7 | 8 | 15* | 22* | [29*] | [36*] | EOC^2^ |
| Clinical assessments |  |  |  |  |  |  |  |  |  |  |  |  |  |  |
| Signs/symptoms | X |  |  |  |  |  |  |  |  |  |  |  |  | x |
| Vital signs | X | x | x | x |  |  |  |  | x^W^ | x^W^ | x^W^ | [x^W^] | [x^W^] | x |
| Physical examination | X | x^o^ |  |  |  |  |  |  | x^W^ | x^W^ | x^W^ | [x^W^] | [x^W^] | x |
| ECG |  | X^o^ |  |  |  |  |  |  |  |  |  |  |  |  |
| Extramedullary involvement | X |  |  |  |  |  |  |  |  |  |  |  |  | X |
| ECOG PS | X | X^o^ |  |  |  |  |  |  | x^W^ | x^W^ | x^W^ | [x^W^] | [x^W^] | X |
| Laboratory assessments |  |  |  |  |  |  |  |  |  |  |  |  |  |  |
| Hematology | X | x | X^SL^ | X^SL^ |  |  |  |  | x^SL,w^ | x^SL,w^ | x^SL,w^ | [x^SL,w^] | [x^SL,w^] | x |
| Basic blood chemistry | X | x |  |  |  |  |  |  | x^SL,w^ | x^SL,w^ | x^SL,w^ | [x^SL,w^] | [x^SL,w^] | x |
| Extended blood chemistry  & coagulation | X |  |  |  |  |  |  |  | x^SL,w^ | x^SL,w^ | x^SL,w^ | [x^SL,w^] | [x^SL,w^] | X |
| Local disease assessment | X |  |  |  |  |  |  |  |  |  |  |  |  | X |
| Central laboratory assessments |  |  |  |  |  |  |  |  |  |  |  |  |  |  |
| Sample collection (BM, PB) | X |  |  |  |  |  |  |  |  |  |  |  |  | x |
| MRD & Disease status | X |  |  |  |  |  |  |  |  |  |  |  |  | X |
| PROs & Health economics |  |  |  |  |  |  |  |  |  |  |  |  |  |  |
| Patient Reported Outcomes | x |  |  |  |  |  |  |  |  |  |  |  |  | X |
| Resource utilization questionnaire | X |  |  |  |  |  |  |  |  |  |  |  |  | X |
| Treatment |  |  |  |  |  |  |  |  |  |  |  |  |  |  |
| ST: High-dose Cytarabine |  | X | X | X |  |  |  |  |  |  |  |  |  |  |
| ST: Mitoxantrone |  |  | x | x |  |  |  |  |  |  |  |  |  |  |
| Safety |  |  |  |  |  |  |  |  |  |  |  |  |  |  |
| Concomitant medications & treatment | X | x | x | x | x | x | x | x | x^w^ | x^W^ | x^W^ | [x^W^] | [x^W^] | x |
| AE assessment | X | x | x | x | x | x | x | x | x^w^ | x^W^ | x^W^ | [x^W^] | [x^W^] | x |
| Pregnancy test  (WOCBP only) |  | x^o^ |  |  |  |  |  |  |  |  |  |  |  |  |
| **Footnotes:**  ^1^ in case of IT day 15 bone marrow blast count >10% or no CR/CRi after the induction therapy cycle  ^2^ includes treatment-free recovery period  * approximate number of day for assessments in weekly intervals (i.e. day given as calculated)  O=to be omitted if done within preceding 48h  SL=Safety lab, values not captured in eCRF  W=to be done in weekly intervals (preferably same day per week) | | | | | | | | | | | | | | |

## Table 3. Detailed Description of Study Visits (Day by Day) consolidation therapy

|  | | **Consolidation Therapy (CT), two cycles** | | | | | | | | | | | | | | | | | | | |
| --- | --- | --- | --- | --- | --- | --- | --- | --- | --- | --- | --- | --- | --- | --- | --- | --- | --- | --- | --- | --- | --- |
| **Phase** | **IT** | **CT cycle 1** | | | | | | | **CT recovery**  **[optional]** | | **End of cycle** | **CT cycle 2** | | | | | | | **CT recovery**  **[optional]** | | **End of cycle** |
| **DAY of Cycle [optional]** | **EOC** | 1 | 2 | 3 | 4 | 8* | 15* | 22* | [29*] | [36*] | EOC^1^ | 1 | 2 | 3 | 4 | 8* | 15* | 22* | [29*] | [36*] | EOC^1^ |
| Clinical assessments |  |  |  |  |  |  |  |  |  |  |  |  |  |  |  |  |  |  |  |  |  |
| Signs/symptoms | x |  |  |  |  |  |  |  |  |  | X |  |  |  |  |  |  |  |  |  | X |
| Vital signs | x | x^O^ |  |  | x | x^W^ | x^W^ | x^W^ | [x^W^] | [x^W^] | x | x^O^ |  |  | x | x^W^ | x^W^ | x^W^ | [x^W^] | [x^W^] | x |
| Physical  examination | x | x^O^ |  |  | x | x^W^ | x^W^ | x^W^ | [x^W^] | [x^W^] | x | x^O^ |  |  | x | x^W^ | x^W^ | x^W^ | [x^W^] | [x^W^] | x |
| ECG |  | x^O^ |  |  |  |  |  |  |  |  | X | x^O^ |  |  |  |  |  |  |  |  | X |
| Extramedullary involvement | X |  |  |  |  |  |  |  |  |  | X |  |  |  |  |  |  |  |  |  | X |
| ECOG PS | X | x^O^ |  |  |  |  |  |  |  |  | x | x^O^ |  |  |  |  |  |  |  |  | x |
| Laboratory assessments |  |  |  |  |  |  |  |  |  |  |  |  |  |  |  |  |  |  |  |  |  |
| Hematology | x | x^o,SL^ |  |  | x^SL^ | x^SL,W^ | x^SL,W^ | x^SL,W^ | [x^SL,W^] | [x^SL,W^] | x | x^o,SL^ |  |  | x^SL^ | x^SL,W^ | x^SL,W^ | x^SL,W^ | [x^SL,W^] | [x^SL,W^] | x |
| Basic blood  chemistry | x | x^o,SL^ |  |  | x^SL^ | x^SL,W^ | x^SL,W^ | x^SL,W^ | [x^SL,W^] | [x^SL,W^] | x | x^o,SL^ |  |  | x^SL^ | x^SL,W^ | x^SL,W^ | x^SL,W^ | [x^SL,W^] | [x^SL,W^] | x |
| Ext. blood chemistry  & coagulation | X | x^o,SL^ |  |  | x^SL^ | x^SL,W^ | x^SL,W^ | x^SL,W^ | [x^SL,W^] | [x^SL,W^] | X | x^o,SL^ |  |  | x^SL^ | x^SL,W^ | x^SL,W^ | x^SL,W^ | [x^SL,W^] | [x^SL,W^] | X |
| Local disease assessment |  |  |  |  |  |  |  |  |  |  |  |  |  |  |  |  |  |  |  |  | X |
| Central laboratory assessments |  |  |  |  |  |  |  |  |  |  |  |  |  |  |  |  |  |  |  |  |  |
| Sample collection  (BM, PB) | x |  |  |  |  |  |  |  |  |  | X |  |  |  |  |  |  |  |  |  | X |
| MRD & Disease status | X |  |  |  |  |  |  |  |  |  | X |  |  |  |  |  |  |  |  |  | X |
| PROS & Health economics |  |  |  |  |  |  |  |  |  |  |  |  |  |  |  |  |  |  |  |  |  |
| Patient Reported Outcomes | X |  |  |  |  |  |  |  |  |  | X |  |  |  |  |  |  |  |  |  | X |
| Resource utilization questionnaire | X |  |  |  |  |  |  |  |  |  | X |  |  |  |  |  |  |  |  |  | X |
| Treatment |  |  |  |  |  |  |  |  |  |  |  |  |  |  |  |  |  |  |  |  |  |
| SOC: Cytarabine |  | x | x | x |  |  |  |  |  |  |  | X | X | X |  |  |  |  |  |  |  |
| Glasdegib/Placebo |  | daily from cycle day 1 to 28 | | | | | | |  |  |  | daily from cycle day 1 to 28 | | | | | | |  |  |  |
| Drug Compliance |  |  |  |  |  |  |  |  |  |  | X |  |  |  |  |  |  |  |  |  | X |
| Safety |  |  |  |  |  |  |  |  |  |  |  |  |  |  |  |  |  |  |  |  |  |
| Concomitant medi- cations & treatment | X | x | x | x | x | x^W^ | x^W^ | x^W^ | [x^W^] | [x^W^] | X | x | x | x | x | x^W^ | x^W^ | x^W^ | [x^W^] | [x^W^] | X |
| AE assessment | X | x | x | x | x | x^W^ | x^W^ | x^W^ | [x^W^] | [x^W^] | X | x | x | x | x | x^W^ | x^W^ | x^W^ | [x^W^] | [x^W^] | X |
| Pregnancy test  (WOCBP only) |  | x |  |  |  |  |  |  |  |  |  | X |  |  |  |  |  |  |  |  | X |
| **Footnotes:**  ^1^ includes treatment-free recovery period of up to 2 weeks if needed  * approximate number of day for assessments in weekly intervals (i.e. day given as calculated)  O=to be omitted if done within preceding 48h  SL=Safety lab, values not captured in eCRF  W=to be done in weekly intervals (preferably same day per week) | | | | | | | | | | | | | | | | | | | | | |

## Table 4. Detailed Description of Study Visits (Day by Day) maintenance therapy

|  | | **Maintenance Therapy (MT), six cycles** | | | | | | | | | | | | |
| --- | --- | --- | --- | --- | --- | --- | --- | --- | --- | --- | --- | --- | --- | --- |
| **Phase** | **CT** | **MT cycle 1** | | **MT cycle 2** | | **MT cycle 3** | | **MT cycle 4** | | **MT cycle 5** | | **MT cycle 6** | | **End of treat-ment** |
| **DAY of Cycle** | **EOC** | 1-27 | 28/ EOC | 1-27 | 28/ EOC | 1-27 | 28/ EOC | 1-27 | 28/ EOC | 1-27 | 28/ EOC | 1-27 | 28/ EOC | EOT |
| Clinical assessments |  |  |  |  |  |  |  |  |  |  |  |  |  |  |
| Signs/symptoms | x |  |  |  |  |  | X |  |  |  |  |  | X | x^o^ |
| Vital signs | x |  | X |  | X |  | x |  | X |  | X |  | x | x^o^ |
| Physical  examination | x |  | x |  | x |  | x |  | x |  | x |  | x | x^o^ |
| ECG | X |  | x |  | x |  | x |  | x |  | x |  | x | x^o^ |
| Extramedullary involvement | X |  |  |  |  |  | x |  |  |  |  |  | x | x^o^ |
| ECOG PS | X |  | x |  | x |  | x |  | x |  | x |  | x | x^o^ |
| Laboratory assessments |  |  |  |  |  |  |  |  |  |  |  |  |  |  |
| Hematology | x |  | X |  | X |  | X |  | X |  | X |  | X | x^o^ |
| Basic blood  chemistry | x |  | x |  | x |  | x |  | x |  | x |  | x | x^o^ |
| Ext. blood chemistry  & coagulation | X |  | x |  | x |  | x |  | x |  | x |  | x | x^o^ |
| Local disease assessment |  |  |  |  |  |  | X |  |  |  |  |  | x | x^o^ |
| Central laboratory assessments |  |  |  |  |  |  |  |  |  |  |  |  |  |  |
| Sample collection  (BM, PB) | x |  |  |  |  |  | x |  |  |  |  |  | x | x^o^ |
| MRD & Disease status | X |  |  |  |  |  | x |  |  |  |  |  | x | x^o^ |
| PROs & Health economics |  |  |  |  |  |  |  |  |  |  |  |  |  |  |
| Patient Reported Outcomes | X |  |  |  |  |  | x |  |  |  |  |  | x | x^o^ |
| Resource utilization questionnaire | X |  |  |  |  |  | X |  |  |  |  |  | X | x^o^ |
| Treatment |  |  |  |  |  |  |  |  |  |  |  |  |  |  |
| Glasdegib / Placebo | (X) | x^D^ | x | x^D^ | x | x^D^ | x | x^D^ | x | x^D^ | x | x^D^ | x |  |
| Drug Compliance | X |  | X |  | X |  | X |  | X |  | X |  | X | x^o^ |
| Safety |  |  |  |  |  |  |  |  |  |  |  |  |  |  |
| Concomitant medi- cations & treatment | X |  | x |  | x |  | x |  | X |  | X |  | X | X |
| AE assessment | X |  | x |  | x |  | x |  | x |  | X |  | X | X |
| Pregnancy test  (WOCBP only) | x |  | x |  | x |  | x |  | x |  | X |  | X | x^o^ |
| **Footnotes:**  D=daily O=to be omitted if done within preceding 48h | | | | | | | | | | | | | | |

## Table 5. Detailed Description of Study Visits (Day by Day) follow-up

|  | | **Safety follow-up (SA) and observational follow-up (FU)** | | | | | | | |  |
| --- | --- | --- | --- | --- | --- | --- | --- | --- | --- | --- |
| **Phase** | **MT** | **Safety  follow-up** | | **Observational follow-up (FU)** | | | | | | **End of study** |
| **Weeks (W)/  Months (M) from EOT [optional]** | EOT | W4 | W8 | M3 | M6 | M9 | M12 | M15 | [3-month intervals starting with M18] | EOS |
| Clinical assessments |  |  |  |  |  |  |  |  |  |  |
| Signs/symptoms | x^O^ | X | X | X | X | X | X | X | X^Y^ | X |
| Vital signs | x^O^ | X | X | X | X | X | X | X | X^Y^ | X |
| Physical examination | x^O^ | X | X | X | X | X | X | X | X^Y^ | X |
| ECG | x^O^ |  |  |  |  |  |  |  |  | X |
| Extramedullary involvement | x^O^ |  |  | X | X | X | X | X | X^Y^ | X |
| Patient Reported Outcomes | x^O^ |  |  | X | X | X | X | X | X^Y^ | X |
| ECOG PS | x^O^ | X | X | X | X | X | X | X | X^Y^ | X |
| Laboratory assessments | x^O^ |  |  |  |  |  |  |  |  |  |
| Hematology | x^O^ | X | X | X | X | X | X | X | X^Y^ | X |
| Basic blood chemistry | x^O^ | X | X | X | X | X | X | X | X^Y^ | X |
| Extended blood chemistry  & coagulation | x^O^ | X | X |  |  |  |  |  |  |  |
| Local disease  assessment |  |  |  | X | X | X | X | X | X^Y^ | X |
| Central laboratory assessments | x^O^ |  |  |  |  |  |  |  |  |  |
| Sample collection  (BM, PB) | x^O^ |  |  | X | X | X | X | X | X^Y^ | X |
| MRD & Disease status | x^O^ |  |  | X | X | X | X | X | X^Y^ | X |
| Safety | x^O^ |  |  |  |  |  |  |  |  |  |
| Concomitant medications  & treatment | x |  |  |  |  |  |  |  |  |  |
| AE assessment | x | X | X |  |  |  |  |  |  |  |
| Pregnancy test  (WOCBP only) | x^O^ | X | X |  |  |  |  |  |  |  |
| **Footnotes:**  O=to be omitted if done within preceding 48h  Y=after 2 years from study day 1, on- site visits are no longer mandatory and may be replaced by contacting the   treating physician or mailing the questionnaire. In this case, no further samples are collected. | | | | | | | | | | |
