# Supplementary material for: Rationale and design of the 2 by 2 factorial design GnG-trial: a randomized phase-III study to compare two schedules of gemtuzumab ozogamicin as adjunct to intensive induction therapy and to compare double-blinded intensive postremission therapy with or without glasdegib in older patients with newly diagnosed AML
Source: Trials. 2021 Nov 3;22:765. doi: 10.1186/s13063-021-05703-w (PMC8564967; doi:10.1186/s13063-021-05703-w)
Supplement: Supplementary file 2 — Additional file 2. SPIRIT Checklist for Trials [file 13063_2021_5703_MOESM2_ESM.pdf]

SPIRIT Checklist for *Trials*

Complete this checklist by entering the page and line numbers where each of the items listed below can be found in your manuscript.

Your manuscript may not currently address all the items on the checklist. Please modify your text to include the missing information. If you are certain that an item does not apply, please state "n/a" and provide a short explanation. **Leaving an item blank or stating "n/a" without an explanation will lead to your manuscript being returned before review.**

Upload your completed checklist as an additional file when you submit to *Trials*. You must reference this additional file in the main text of your protocol submission. The completed SPIRIT figure must be included within the main body of the protocol text and can be downloaded here: http://www.spirit-statement.org/schedule-of-enrolment-interventions-and-assessments/

In your methods section, please state that you used the SPIRIT reporting guidelines, and cite them as:

Chan A-W, Tetzlaff JM, Gøtzsche PC, Altman DG, Mann H, Berlin J, Dickersin K, Hróbjartsson A, Schulz KF, Parulekar WR, Krleža-Jerić K, Laupacis A, Moher D. SPIRIT 2013 Explanation and Elaboration: Guidance for protocols of clinical trials. BMJ. 2013;346:e7586

		Reporting Item	Page and Line Number	Reason if not applicable
**Administrative information**				
Title	#1	Descriptive title identifying the study design, population, interventions, and, if applicable, trial acronym	Page 1, line 1.	

Trial registration	#2a	Trial identifier and registry name. If not yet registered, name of intended registry	Page 4 Line 120-121.	
Trial registration: data set	#2b	All items from the World Health Organization Trial Registration Data Set	Page 4 Line 122.	
Protocol version	#3	Date and version identifier	Page 1 Line 28.	
Funding	#4	Sources and types of financial, material, and other support	Page 2 Line 71 and 72 and page 19 line 534-539. A letter from Pfizer was attached.	
Roles and responsibilities: contributorship	#5a	Names, affiliations, and roles of protocol contributors	Pages 1 and 2 Lines: 30-66 and page 19 and page 19 and 20 lines 554-555.	
Roles and responsibilities: sponsor contact information	#5b	Name and contact information for the trial sponsor	Page 19 and 20 lines 554-555.	

Roles and responsibilities: sponsor and funder	#5c	Role of study sponsor and funders, if any, in study design; collection, management, analysis, and interpretation of data; writing of the report; and the decision to submit the report for publication, including whether they will have ultimate authority over any of these activities	Page 19 and 20 lines 554-555.	
Roles and responsibilities: committees	#5d	Composition, roles, and responsibilities of the coordinating centre, steering committee, endpoint adjudication committee, data management	The Coordinating Investigator should ensure that all persons assisting with the trial are adequately informed about the protocol and any amendments, the trial treatments, and their trial-related duties and functions.<br>The Coordinating Investigator should maintain a list of investigators and other appropriately qualified persons to whom he or she has delegated significant trial-related duties (Log of Staff).<br>The investigators should support monitoring, auditing and inspections.<br>Before the start of the trial, the trial protocol, informed consent document, and any other appropriate documents are submitted to the independent Ethics Committee (EC) as well as to the competent federal authority (BfArM). A written favorable vote of the EC and an (implicit) approval by the competent higher federal authority are a prerequisite for initiation of the clinical trial. The statement of EC should contain the title of the trial, the trial code, the trial site, and a list of reviewed documents. It must mention the date on which the decision was made and must be officially signed by a committee member.	

		team, and other individuals or groups overseeing the trial, if applicable (see Item 21a for data monitoring committee)	Before the first patient is enrolled in the trial, all ethical and legal requirements must be met. All planned substantial changes (see §10, (1) of German GCP-Regulation) are to be submitted to EC and the competent federal authority in writing as amendments. They have to be approved by the EC and the competent federal authority.<br><br>The Coordinating Investigator or the NCT Trial Center, and if applicable the investigator(s) are keeping a record of all communication with the EC and the regulatory authorities.<br><br>See item 21a for monitoring committee.	
**Introduction**				
Background and rationale	#6a	Description of research question and justification for undertaking the trial, including summary of relevant studies (published and unpublished) examining benefits and harms for each intervention	Pages 5-8, lines 125-221.	
Background and rationale: choice of comparators	#6b	Explanation for choice of comparators	Pages 7 and 8, lines 180-221.<br>**Justification for the use of Placebo**<br>In this study glasdegib or placebo is added to standard of care chemotherapy during consolidation therapy and as single agent during maintenance to investigate if the addition of glasdegib is beneficial. Patients of the control group are taking placebo in addition to active	

			standard treatment, i.e. they receive the approved and recommended standard regimen. Hence, patients of the control have no disadvantage as compared to patients outside the study. Based on this the use of placebo is justified and necessary to achieve highest scientific validity.	
Objectives	#7	Specific objectives or hypotheses	Page 8 Lines 224-227.<br><br>**Primary Objectives**<br><br>The primary objectives of the present trial are:<br><br>• To assess clinical efficacy of sequential or one-dose gemtuzumab ozogamicin as adjunct to induction therapy in older patients with newly diagnosed AML. Clinical efficacy is determined by MRD-negativity after induction therapy.<br><br>To assess clinical efficacy of glasdegib as adjunct to 2 months consolidation and as single agent 6 months maintenance therapy in older patients with newly diagnosed AML. Clinical efficacy is determined by event-free survival (EFS) defined as the time from randomization to time until one of the following events, whichever occurs first: a) failure to obtain complete remission (CR) or complete remission with incomplete hematological recovery (CRi), b) relapse from CR/CRi for patients with induction success or c) death from any cause. Patients without an applicable event are censored on the last date of follow-up.<br><br>**The secondary objectives of the present trial are:**<br><br>• Evaluation of efficacy based on complete remission rate (CRR) and overall survival (OS).<br><br>• Evaluation of relapse-free survival (RFS), defined as the time from achievement of a CR/CRi after randomization to time of recurrence of the disease or death from any cause, whatever occurs first. Patients without the event are censored on the last date of follow-up.<br><br>• Assessment of patient reported outcomes (PRO, including quality of life (QoL)) after induction, consolidation and maintenance therapy and after at least two years	

			• Evaluation of safety based on duration of neutropenia and leukopenia, incidence of infection, duration of initial hospitalization.<br><br>• Cost-effectiveness analysis of the four different treatment schedules from health care payer´s perspective.<br><br>• Budget impact analysis of introducing effective treatment schedule(s) in everyday clinical practice.<br><br>• Mapping the EORTC QLQ-C30 cancer specific instrument to the SF-36 generic instrument for older patients with newly diagnosed AML in Germany.	
Trial design	#8	Description of trial design including type of trial (eg, parallel group, crossover, factorial, single group), allocation ratio, and framework (eg, superiority, equivalence, non-inferiority, exploratory)	Page 8 Lines 224-227.<br><br>The study is a multicenter, randomized phase III trial with MRD after induction therapy and event-free survival as primary endpoints. The two research questions are addressed in a 2 by 2 factorial design. Patients are upfront randomized for the two induction schedules (GO-147 versus GO-1) and for glasdegib or placebo (double blinded) as adjunct to consolidation therapy and as single agent 6 months maintenance therapy in a 1:1:1:1 ratio. Chemotherapy backbone for induction therapy is standard 7+3 with cytarabine 200mg/m² continuously day 1 to day 7, daunorubicin 60mg/m² days 1, 2 and 3 and for consolidation therapy intermediate dose cytarabine (1g/m², bi-daily, days 1,2,3). The trial is designed to gain evidence of anti-leukemic activity of gemtuzumab ozogamicin and glasdegib in older patients with newly diagnosed acute myeloid leukemia.	
**Methods: Participants, interventions, and outcomes**				
Study setting	#9	Description of study settings (eg, community clinic, academic	Pages 1 and 2 line 41-66.<br><br>Multi-centre trial conducted in several German university medical centres	

		hospital) and list of countries where data will be collected. Reference to where list of study sites can be obtained		
Eligibility criteria	#10	Inclusion and exclusion criteria for participants. If applicable, eligibility criteria for study centres and individuals who will perform the interventions (eg, surgeons, psychotherapists)	Table 3, page 27, line 736.	
Interventions: description	#11a	Interventions for each group with sufficient detail to allow replication, including how and when they will be administered	Page 9 and 10, lines 239-285 and Figure 1<br><br>**Induction therapy:**<br><table><tr><th>Arm</th><th>Type</th><th>Drug</th><th>Administration</th><th>Days</th></tr><tr><td>all</td><td>SOC</td><td>Cytarabine</td><td>200mg/m², i.v. continuously</td><td>1 to 7</td></tr><tr><td>all</td><td>SOC</td><td>Daunorubicin</td><td>60mg/m², i.v. 1h infusion</td><td>1, 2,3</td></tr><tr><td>GO-147</td><td>IMP</td><td>Gemtuzumab Ozogamicin</td><td>3mg/m² (cap at 5mg absolute), i.v. 2h infusion (Mylotarg®).</td><td>1, 4, 7</td></tr><tr><td>GO-1</td><td>IMP</td><td>Gemtuzumab Ozogamicin</td><td>3mg/m² (cap at 5mg absolute), i.v. 2h infusion (Mylotarg®)</td><td>1</td></tr></table>	

<table>
<tr><td></td><td></td><td></td><td>
<table>
<tr><th>Consolidation therapy (2 cycles): Arm</th><th>Type</th><th>Drug</th><th>Administration</th><th>Days</th></tr>
<tr><td>All</td><td>SOC</td><td>Cytarabine</td><td>1g/m², i.v. 3h infusion bi-daily</td><td>1, 2, 3</td></tr>
<tr><td>Experimental</td><td>IMP</td><td>Glasdegib</td><td>100mg, tablet</td><td>1 to 28</td></tr>
<tr><td>Standard</td><td>NIMP</td><td>Placebo</td><td>100mg, tablet</td><td>1 to 28</td></tr>
</table>
</td><td></td></tr>
<tr><td>Interventions: modifications</td><td>#11b</td><td>Criteria for discontinuing or modifying allocated interventions for a given trial participant (eg, drug dose change in response to harms, participant request, or improving / worsening disease)</td><td>Table 1 and 2, pages 25 and 26.</td><td></td></tr>
<tr><td>Interventions: adherance</td><td>#11c</td><td>Strategies to improve adherence to intervention protocols, and any procedures for monitoring</td><td>Page 10, lines 273-276.
Gemtuzumab ozogamicin is administered on site as intravenous infusion.
For glasdegib/placebo, all patients maintain patient dosing diaries throughout the study recording dates of administration and all regular, missed, changed, or delayed doses.
Patients are required to return all bottles, unused study drug and the patient dosing diary, after each cycle and at EOT visit for compliance assessment and drug accountability. The number of tablets returned by the patient at the end of the cycle is counted, documented and recorded. Dates of drug intake and all missed doses must be recorded. Bottles (empty or containing unused tables) and dosing diaries are to be returned.</td><td></td></tr>
</table>

		adherence (eg, drug tablet return; laboratory tests)		
Interventions: concomitant care	#11d	Relevant concomitant care and interventions that are permitted or prohibited during the trial	During and following a patient's participation in the trial, the investigator should ensure that adequate medical care is provided to a patient for any adverse event, including clinically significant laboratory findings. The investigator should inform a patient when medical care is needed for intercurrent illness(es) of which the investigator becomes aware.<br><br>**Prior and Concomitant Medication / Non-Drug Treatment**<br><br>All concomitant medications and treatments must be recorded in the CRF. Any prior treatment received within 28 days prior to start of study treatment (including hematopoietic growth factor receptor agonists: erythropoietin, (G-CSF), romiplostim, eltrombopag) are to be recorded in the CRF.<br><br>Every concomitant treatment, blood products, any transfusion (red blood cells or platelets), growth factors, as well as interventions, required by the patients during the active study treatment (and up to 28 days following last study drug administration or until initiation of another anti-cancer treatment) and the reason for its administration must be recorded on the CRF.<br><br>All concomitant medications the patient is currently receiving must be reviewed by the Investigator prior to enrollment.<br><br>Restricted or Prohibited Concomitant Medications<br><br>The following medications are not allowed during the active study consolidation and maintenance period:<br><br>• Erythropoietin or darbepoietin;<br><br>• Thrombopoietin receptor agonists (e.g., eltrombopag, romiplostim);	

			• Hydroxyurea or other anti-cancer agents (e.g., tacrolimus, hormones, cytokines, etc.);<br><br>• Investigational agents for the treatment of hematologic malignancies;<br><br>• Immunosuppressant agents (e.g., cyclosporine);<br><br>• CYP3A4/5 Inducers: glasdegib metabolism may be induced when taking CYP3A4/5 inducers, resulting in reduced plasma concentrations. The impact of CYP3A4/5 inducers on glasdegib pharmacokinetics has not been studied in the clinic. Therefore co-administration of glasdegib with any of the following and other moderate/strong CYP3A4/5 inducers is not permitted (unless approved by the principle investigator or the scientific coordinator) from study entry until study treatment discontinuation (avasimibe, mitotane, phenytoin, enzalutamide, semagacestat, bosentan, genistein, thioradazine, nafcillin, modafinil, carbamazepine, phenobarbital, phenytoin, rifampin, rifabutin, rifapentine, St. John’s Wort). In case of uncertainty whether a concomitant medication is contraindicated, the principle investigator or the scientific coordinator should be contacted.<br><br>The following medications have use restrictions during the active study consolidation and maintenance period:<br><br>• Aspirin in doses exceeding 300 mg per day is not permitted.<br><br>• Oral anticoagulation with warfarin is not recommended if alternative medication (e.g., low molecular weight heparin) can be substituted as per investigator judgment. If warfarin is indispensable, frequent monitoring of the International Normalized Ratio (INR) is recommended and the dosage of oral anticoagulant should be adjusted as needed.<br><br>• CYP3A4/5 Inhibitors: In vitro studies with human liver microsomes and recombinant CYP enzymes indicated that glasdegib metabolism is primarily mediated by the drug-metabolizing enzyme CYP3A4/5. Clinically, there is likelihood that glasdegib plasma concentrations may be increased in the presence of co-administered inhibitors of the CYP3A4/5 enzymes. In a healthy volunteer study, ketoconazole, a potent CYP3A4/5 inhibitor, produced a 2.4-fold increase in plasma exposure and a 1.4-fold increase in peak plasma concentration of glasdegib. Therefore, a	

			potential exists for drug-drug interactions with CYP3A4/5 inhibitors, and co-administration of glasdegib in combination with moderate/strong CYP3A4/5 inhibitors is not recommended. Selection of concomitant medication with no or minimal CYP3A4/5 inhibition potential is recommended. Moderate/strong CYP3A4/5 inhibitors should be used with caution and only if considered medically necessary.<br><br>• Corticosteroids: Chronic, systemic corticosteroid use for palliative, supportive purpose or for other baseline disease is not permitted. Acute administration, topical applications, inhaled sprays, eye drops or local injections of corticosteroids are allowed.<br><br>• Surgery: Caution is advised on theoretical grounds for any surgical procedures during the study. The appropriate interval of time between surgery and glasdegib required to minimize the risk of impaired wound healing and bleeding has not been determined. Stopping glasdegib is recommended at least 7 days prior to surgery. Post-operative reinitiation of glasdegib treatment is basically at the Investigator's discretion but requires approval of the principle investigator or the scientific coordinator and should be based on a clinical assessment of satisfactory wound healing and recovery from surgery.	
Outcomes	#12	Primary, secondary, and other outcomes, including the specific measurement variable (eg, systolic blood pressure), analysis metric (eg, change from baseline, final value, time to event), method	Pages 11 and 12, lines 312-342.<br><br>**See #20a.**	

<table>
<tr><td></td><td></td><td>of aggregation (eg, median, proportion), and time point for each outcome. Explanation of the clinical relevance of chosen efficacy and harm outcomes is strongly recommended</td><td></td><td></td></tr>
<tr><td>Participant timeline</td><td>#13</td><td>Time schedule of enrolment, interventions (including any run-ins and washouts), assessments, and visits for participants. A schematic diagram is highly recommended (see Figure)</td><td>

Overall duration The study runs until the last patient being alive has been observed for at least 2 years. Assuming 2 years recruitment, the follow-up of the first patient lasts up to 4 years.

The study consist of the following consecutive phases:

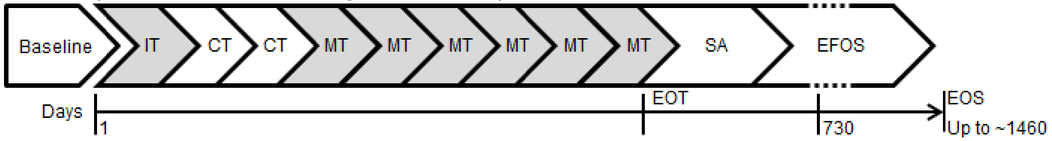

BL Baseline: up to 14 days prior to inclusion
IT Induction therapy: 1 cycle á 7 days followed by a recovery period with no treatment for 3-5 weeks (28-82 days in total)
CT Consolidation therapy: 2 cycles á 28 days each consisting of 3 days chemo therapy, 24 days glasdegib and 1 days with no treatment, followed by up to 2 weeks recovery period if needed (2x 28-42 days = 56-84 days in total)
MT Maintenance therapy: 6 cycles á 28 days (168 days in total)
EOT End of treatment: the last day of the last maintenance therapy
SA Safety Follow-up: 8 weeks safety follow up (56 days in total)
EFOS Event free and overall-survival: observational follow-up for event-free survival and overall survival at least until the end of the whole study. After achieving an observation period of 2 years counted from day 1, the follow-up may be performed by contacting the treating physician instead of in house-visits. (At least 418- 1106 days in total)
EOS End of study: The study ends for all patients when the last patient being included and alive has been followed for at least 730 days (2 years) counting from this patient's day 1.

***See figure attached.</td><td></td></tr>
</table>

Sample size	#14	Estimated number of participants needed to achieve study objectives and how it was determined, including clinical and statistical assumptions supporting any sample size calculations	Pages 13-15, lines 371-407.<br><br>The trial incorporates two primary endpoints, namely the short-term endpoint MRD-negativity (MRD) and the long-term endpoint event-free survival (EFS).<br><br>The short-term evaluation involves a comparison of MRD rates between the experimental arm GO-147 and the control arm GO-1. The null hypothesis is H0ST: pGO-147 = pGO-1. Assuming an MRD of 45% for the GO-147 arm and an MRD of 20% for the GO-1 arm, as well as a 3% dropout rate, a total number of 252 evaluable patients are needed to reject the null hypothesis at a two-sided significance level of 2.5% with a power of approximately 85% using a chi-squared test (details of the calculation are provided in Sections 10.3.2 and 10.3.4). It is assumed that using a generalized linear mixed model adjusting for center, age and ECOG PS yields an increased power due to part of the variance being explained by confounders.<br><br>The long-term evaluation involves a two group comparison of EFS between the experimental arm HiDAC + glasdegib and the control arm HiDAC + placebo. The null hypothesis is H0LT: There is no difference in EFS between the HiDAC + glasdegib arm as compared to the HiDAC + placebo arm. Assuming an EFS of 45% for the experimental arm and an EFS of 70% for the control arm, as well as a 5% dropout rate, a total number of 224 evaluable patients are needed to reject the null hypothesis at a significance level of 2.5% with a power of approximately 85% (details of the calculation are provided in Sections 10.3.3 and 10.3.4). As for the short-term endpoint, it is assumed that using a Cox regression model adjusting for center, age and ECOG PS (0 / >0) yields an increased power due to part of the variance being explained by confounders. Calculations were performed using the software ADDPLAN v6.1.<br><br>**General Considerations and Test Hypotheses**<br><br>In the following, we illustrate the considerations on the choice of the sample size for the research questions GO-147 vs. GO-1 and HiDAC + glasdegib vs. HiDAC + placebo within a 2x2 factorial design. The two research questions result in four treatment arms:<br><br>A) GO-147 & HiDAC + glasdegib	

			B) GO-1 & HiDAC + glasdegib<br><br>C) GO-147 & HiDAC + placebo<br><br>D) GO-1 & HiDAC + placebo<br><br>The trial incorporates two primary endpoints, namely MRD-negativity (yes/no) defined as absence of leukemic cells at the end of the induction therapy<br><br>and event-free survival (EFS) defined as the time from randomization until one of the following events occurs first:<br><br>(i) failure to obtain CR or CRi,<br><br>(ii) relapse from CR/CRi or<br><br>(iii) death.<br><br>Within this trial, it is hypothesized that GO-147 leads to an increased MRD-negativity rate as compared to GO-1, and that HiDAC + glasdegib leads to an improved EFS as compared to HiDAC + placebo. It is assumed that there is no (relevant) treatment interaction.<br><br>The assessment of the two null hypotheses H0ST for the short-term endpoint MRD-negativity and H0LT for the long-term endpoint EFS requires an adjustment of the (two-sided) local significance levels αST and αLT in order to control the family-wise error rate in the strong sense at a global two-sided significance level of α=0.05. Therefore, the Bonferroni-Holm approach is used, being uniformly more powerful than the Bonferroni approach without requiring any additional assumptions.<br><br>**Considerations for the Short-Term Endpoint MRD-Negativity**<br><br>The proportion of patients who achieve a complete remission (CR/CRi) of acute myeloid leukemia (AML) after treatment is assumed to be 70% irrespective of the treatment group [14]. For a	

patient from one of the GO-1 groups B/D with a CR/CRi, the probability to be MRD-negative is assumed to be 20% [19]. Hence, the overall MRD-negative rate for patients from one of the two GO-1 groups is assumed to be

pGO-1=0.7 x 0.2=0.14.

Regarding the MRD-negativity rate in the GO-147 patients with a CR/CRi, 4 different scenarios were assumed during the planning phase, namely 50%, 45%, 40% and 35%. Accordingly, the MRD-negativity rate is assumed to be either

- pGO-147=0.7 x 0.5 = 0.35,
- pGO-147=0.7 x 0.45 = 0.315,
- pGO-147=0.7 x 0.4 = 0.28 or
- pGO-147=0.7 x 0.35 = 0.245

The dropout rate for the assessment of the short-term endpoint is assumed to be 3%.

10.3.3 Considerations for the Long-Term Endpoint EFS

Since the proportion of patients who achieve a CR or CRi of acute myeloid leukemia (AML) after treatment is assumed to be 70% irrespective of the treatment group, 30% of all patients experience the event “failure to obtain CR or CRi” regardless of the treatment. For those patients from the “HiDAC only” arms C and D with a CR or CRi, it is assumed that a proportion of 70% either relapse or die two years after randomization. Hence, the proportion of patients from arms C and D with an event after two years, πHiDAC, amounts to

πHiDAC = 0.3 +(0.7 x 0.7)=0.3+0.49=0.79.

Regarding the proportion of patients from the glasdegib groups A and B who either experience a relapse or die within two years after randomization but have achieved a complete remission before, 4 different scenarios were assumed during the planning phase of the trial, namely 35%,

			40%, 45% and 50%. Accordingly, the following scenarios for the event rate πHiDAC+GD after two years are considered:<br><br>• πHiDAC+GD =0.3 + (0.7 x 0.35) = 0.3 + 0.245=0.545<br><br>• πHiDAC+GD =0.3 + (0.7 x 0.4) = 0.3 + 0.28=0.58<br><br>• πHiDAC+GD =0.3 + (0.7 x 0.45) = 0.3 + 0.315=0.615<br><br>• πHiDAC+GD =0.3 + (0.7 x 0.5) = 0.3 +0.35 =0.65<br><br>A dropout rate of 3% is expected for the assessment of the short-term endpoint, which is induced by a proportion of patients for which a MRD measurement cannot be conducted due to a lack of a leukemia-associated phenotype. For the long-term endpoint EFS, a dropout rate of 5% is expected 2 years after randomization and dropout times are assumed to be exponentially distributed. The two distinct dropout probabilities are assumed to be uncorrelated.<br><br>Furthermore, the assumed accrual time amounts to 24 months, and the follow-up time is assumed to be 24 months as well.<br><br>**Required Sample Sizes**<br><br>In the following, the required total sample sizes are displayed for the assessments of the short- and long-term endpoint. The required sample sizes are based on the assumptions in the previous Sections. For the comparison of the short-term endpoint, MRD-negativity, the sample size calculation is based on the comparison by the chi-squared test, while for the comparison of the long-term endpoint, EFS, the log-rank test is applied with the sample size formula by Schoenfeld [47]. Sample sizes were determined for a power of 1-β=0.8, 0.85, and 0.9, respectively. The required total sample sizes for the Bonferroni approach are presented in Table 5.<br><br>Assumptions: pGO-1=0.14, πHiDAC=0.79, αST= αLT=0.025. Dropout rates of 3% for the short-term endpoint and 5% for the long-term endpoint are already incorporated.<br><br>Based on the Bonferroni approach with an aspired power of 1-β=0.85 for both hypotheses, and assuming that pGO-147=0.315 and πHiDAC+GD=0.615, a total sample size of N=max(252,	

			224)=252 patients needs to be randomized. Using the Bonferroni-Holm approach to control for multiple testing yields a further increase in power.	
Recruitment	#15	Strategies for achieving adequate participant enrolment to reach target sample size	Page 8, lines 229-230.<br><br>Recruitment and treatment of patients should be performed in 25 or more centers to recruit the intended number of patients. Expecting a number of at least 5 eligible patients per year and center, approximately 2 years are required to recruit the intended number of patients.	
**Methods: Assignment of interventions (for controlled trials)**				
Allocation: sequence generation	#16a	Method of generating the allocation sequence (eg, computer-generated random numbers), and list of any factors for stratification. To reduce predictability of a random sequence, details of any planned restriction (eg, blocking) should be provided in a separate	Pages 8 and 9, lines 228-238.<br><br>The informed consent has to be signed before enrollment into the study i.e. it must be signed prior to any trial-related procedures including initiation of therapy.<br><br>Each patient having signed informed consent and meeting all inclusion criteria must be registered. Prior to this, each patient intended to be registered must be allotted a screening number by the study site (usually ascending numbers beginning from 1).<br><br>Patients are registered through the eCRF (or per fax registration in case of technical failures) and the unique patient ID (PAT-ID) is assigned via the registration process. The PAT-ID is composed of the aforementioned screening number and the site number.<br><br>Following registration the patient is randomized into one of the study arms and is allotted to a randomization number (Rand-No) in addition to the PAT-ID. Randomization is done using a centralized web-based tool (www.randomizer.at) by which randomization for double-blind clinical trials can easily be handled.<br><br>Patients withdrawn from the trial retain their identification codes (Rand-No and/or PAT-ID, if already given). New patients must always be allotted a new identification code.	

		document that is unavailable to those who enrol participants or assign interventions	Eligible patients are randomized in a concealed fashion to one of the four treatment arms in a 1:1:1:1 ratio. Randomization is performed stratified by age (≤70 years vs. >70 years) and ECOG performance status (ECOG PS = 0 vs. ECOG PS >0), both of which are assumed to be the most important prognostic factors. Block randomization with varying block length is performed to achieve balanced group sizes per stratum. Due to the small number of expected patients per center, randomization is not stratified per center in order to avoid the risk of an unbalanced number of patients between the treatment arms. Instead, "center" is taken into account as a random factor in the statistical model (see Section 10.5 for details).<br><br>Study–medication tablets (glasdegib/placebo) are blinded to patients and investigators. At overall study end, patients will be informed by authorized and unblinded study personnel, which treatment they had been administered during the study.<br><br>For all other trial personnel, including the biometricians, patient treatment shall remain blinded from the time of randomization until final database lock.<br><br>At the end of the study and after data verification and database lock, the assigned blinded codes are broken for the final analysis of study data.<br><br>Detailed instructions on randomization, blinding and breaking the blind are distributed to the respective authorized personnel prior to the start of the study.	
Allocation concealment mechanism	#16b	Mechanism of implementing the allocation sequence (eg, central telephone; sequentially numbered, opaque, sealed envelopes),	**See #16a.**	

		describing any steps to conceal the sequence until interventions are assigned		
Allocation: implementation	#16c	Who will generate the allocation sequence, who will enrol participants, and who will assign participants to interventions	Page 8, line236-236.<br><br>Randomization is done using a centralized web-based tool (www.randomizer.at) by which randomization for double-blind clinical trials can easily be handled. Patient will be enrolled by the investigators of the initiated centers.	
Blinding (masking)	#17a	Who will be blinded after assignment to interventions (eg, trial participants, care providers, outcome assessors, data analysts), and how	Study–medication tablets (glasdegib/placebo) are blinded to patients and investigators. At overall study end, patients will be informed by authorized and unblinded study personnel, which treatment they had been administered during the study.<br><br>For all other trial personnel, including the biometricians, patient treatment shall remain blinded from the time of randomization until final database lock.<br><br>At the end of the study and after data verification and database lock, the assigned blinded codes are broken for the final analysis of study data.<br><br>Detailed instructions on randomization, blinding and breaking the blind are distributed to the respective authorized personnel prior to the start of the study.	
Blinding (masking):	#17b	If blinded, circumstances under which	If it is medically imperative to know what trial medication the patient is receiving, the investigator or authorized medical staff should break the blind of the respective patient. The investigator or the person who breaks the blind must record the date and the reasons for doing so in the online	

emergency unblinding		unblinding is permissible, and procedure for revealing a participant's allocated intervention during the trial	randomization tool (randomizer.at), the eCRF and in the patient's medical record. Whenever possible, the CI and/ or the sponsor should be contacted before the blind is broken.<br><br>The procedure of breaking the blind using randomizer.at is described in a separate document that is handed out prior to initiation of the respective clinical site.	
**Methods: Data collection, management, and analysis**				
Data collection plan	#18a	Plans for assessment and collection of outcome, baseline, and other trial data, including any related processes to promote data quality (eg, duplicate measurements, training of assessors) and a description of study instruments (eg, questionnaires, laboratory tests) along with their	Page 13, lines 354-363.<br><br>The data collection is performed using an eCRF. Data collection using the eCRF can only be done by authorized persons. All study data are password-protected. The eCRF provides several checks for completeness and consistency. Each entry or change of data is tracked with name and exact date (audit trail). When data has been entered, reviewed, edited and Source Data Verification (SDV) performed, the investigator is notified to sign the eCRF electronically as per agreed project process, and data is locked to prevent further editing. A copy of the eCRF is to be archived at the study site.<br><br>All findings including clinical and laboratory data is documented by the investigator or an authorized member of the study team in the patient's medical record and in the eCRF. The investigator is responsible for ensuring that all sections of the eCRF are completed correctly and that entries can be verified by source data. The eCRF has to be filled out according to the specified eCRF Completion Guidelines.<br><br>PRO questionnaires are paper-based, are completed by patients and serve as source data. Upon completion questionnaires (apart from SF-36, see below) are returned (e.g. mailed back or collected by the monitor) to central unit for Quality of Life & Patient-Reported Outcomes (see page 2 "Responsibilities"). The questionnaires are then recorded using the TELEFORM® system (Cardiff) and undergo a computer assisted manual verification. Derived data sets, combining eCRF	

		reliability and validity, if known. Reference to where data collection forms can be found, if not in the protocol	and PRO data are produced at time points of analyses. The link between the questionnaires and the eCRF is maintained by a combination of a unique number for each questionnaire and the patient-ID (PA T-ID) which is recorded in the eCRF.<br><br>Health care resource utilization questionnaires and the SF-36 questionnaire are paper-based and self-administered by the patients. Upon completion, the questionnaires are returned (e.g. mailed back or collected by the monitor) to the Division of Health Economics. The questionnaires are recorded electronically and validated by a second person to ensure accuracy in the data capturing. Derived data sets are merged with relevant queries from the eCRF. The link between the health economic questionnaires and the eCRF is maintained by a combination of a unique number for each questionnaire and the patient-ID (PAT-ID) which is recorded in the eCRF.	
Data collection plan: retention	#18b	Plans to promote participant retention and complete follow-up, including list of any outcome data to be collected for participants who discontinue or deviate from intervention protocols	**Data handling**<br><br>Data entries undergo an automated check for plausibility and consistency. In case of implausibility, 'warnings' are produced. A responsible investigator is obliged either to correct the implausible data or to confirm its authenticity and to give an appropriate explanation. If not corrected, the data are flagged, enabling a convenient check of all questionable entries. The responsible monitor checks all flagged data and generates questions ("queries") that are sent back to the responsible investigator. The investigator has to resolve all 'discrepancies'.<br><br>Further checks for plausibility, consistency, and completeness of data are performed during and after completion of the study. Queries are generated on the basis of these checks, combined with a visual control by a responsible monitor/data manager.<br><br>All missing data or inconsistencies are reported back to the sites and clarified by the responsible investigator. If no further corrections are to be made in the trial database it is declared closed and used for statistical analysis.<br><br>All data management activities are done according to the current Standard Operating Procedures (SOPs).	

Data management	#19	Plans for data entry, coding, security, and storage, including any related processes to promote data quality (eg, double data entry; range checks for data values). Reference to where details of data management procedures can be found, if not in the protocol	**Storage**<br><br>According to legal obligations (§13 of the German GCP-Regulation) all important documents (e.g. CRFs) collected within the scope of this trial are to be archived for at least 10 years after its termination. The trial documents will be destroyed within one and a half year after the end of this retention period.<br><br>The investigator(s) archive all trial data (source data and Investigator Site File (ISF) including Patient Identification List and relevant correspondence) according to the Section 4.9 of the ICH Consolidated Guideline on GCP (E6) and to local law or regulations. The Patient Identification List is archived for at least 15 years after trial termination.<br><br>If the investigator relocates, retires, or for any reason withdraws from the study, the NCT Trial Center should be prospectively notified. The study records must be transferred to an acceptable designee, such as another investigator, another institution, or to the NCT Trial Center. The investigator must obtain CIs written permission before disposing of any records, even if archiving requirements have been met.<br><br>**Confidentiality**<br><br>The data obtained in the course of the trial are treated pursuant to the General Data Protection Regulation (EU-DSGVO, EU 2016/679) and the Data Protection Law of the Federal State (Landesdatenschutzgesetz), and the § 40 (2a) AMG.<br><br>During the clinical trial, patients are identified solely by means of an individual identification code (Patient ID). Storage of trial findings on a computer is done in accordance with local data protection law and handled in strictest confidence. For protection of these data, organizational procedures are implemented to prevent distribution of data to unauthorized persons. The appropriate regulations of local data legislation are fulfilled in its entirety.<br><br>The patient consents in writing to relieve the investigator from his/her professional discretion in so far as to allow inspection of original data for monitoring purposes by health authorities and	

			authorized persons (inspectors, monitors, auditors). Authorized persons may inspect the patient-related data collected during the trial, ensuring the data protection law.<br><br>The investigator has to maintain a patient identification list (Patient IDs with the corresponding patient names) to enable records to be identified.<br><br>Patients who did not consent to circulate their pseudonymized data must not be included into the trial.	
Statistics: outcomes	#20a	Statistical methods for analysing primary and secondary outcomes. Reference to where other details of the statistical analysis plan can be found, if not in the protocol	Pages 14 and 15, lines 372-407.<br><br>**Research Hypothesis**<br><br>This trial addresses two research questions:<br><br>1. Does GO-147 lead to an increased MRD-negativity rate as compared to GO-1?<br><br>2. Does high-dose cytarabine (HiDAC) + glasdegib lead to improved event-free survival as compared to HiDAC + placebo?<br><br>For the short-term endpoint (1) MRD-negativity, the hypotheses are as follows:<br><br>H0ST-: there is no difference regarding the MRD-negativity rate for patients receiving GO-147 (pGO-147) as compared to patients receiving GO-1 (pGO-1) during induction therapy, i.e. pGO-147= pGO-1<br><br>H1ST-: there is a difference regarding the MRD-negativity rate for patients receiving GO-147 as compared to the rate for patients receiving GO-1 during induction therapy, i.e. pGO-147 ≠ pGO-1<br><br>For the long-term endpoint (2) event-free survival, the hypotheses are as follows:<br><br>H0LT-: there is no difference regarding event-free survival for patients receiving HiDAC + glasdegib as compared to patients receiving HiDAC + placebo during consolidation therapy<br><br>H1LT-: there is a difference regarding event-free survival for patients receiving HiDAC + glasdegib as compared to patients receiving HiDAC + placebo during consolidation therapy	

			**Analysis of variables**<br><br>**Primary Endpoints and Primary Estimands**<br><br>For the gemtuzumab ozogamicin primary objective, the primary estimand according to the ICH-E9 (R1) addendum is defined as:<br><br>Treatment: GO-147 (experimental arm) compared to GO-1 (control arm)<br><br>Population: all patients fulfilling the in- and exclusion criteria<br><br>Variable: MRD-negativity (MRD) defined as absence of leukemic cells at the end of the induction therapy assessed by flow-cytometry.<br><br>Post-randomisation events: if MRD-negativity cannot be measured, the outcome will be imputed (hypothetical strategy; see also Section 10.5.3), the outcome of patients who drop out of the study before MRD measurement will be imputed (hypothetical strategy; see also Section 10.5.3), changes in treatment, or discontinuation of treatment will be ignored (treatment policy strategy), any-cause death before MRD measurement will be regarded as MRD-positive (composite strategy).<br><br>Summary measure: Odds ratio for the endpoint MRD-negativity between the two treatment arms<br><br>For the glasdegib primary objective, the primary estimand is defined as:<br><br>Treatment: HiDAC + glasdegib (experimental arm) compared to HiDAC + placebo (control arm)<br><br>Population: all patients fulfilling the in- and exclusion criteria<br><br>Variable: Event-free survival (EFS) defined as the time from randomization to time until one of the following events, whichever occurs first: a) failure to obtain complete remission (CR) or complete remission with incomplete hematological recovery (CRi), b) relapse from CR/CRi for patients with induction success or c) death from any cause.	

			Post-randomisation events: death from any cause is incorporated into the variable definition (composite strategy); changes in treatment and termination of treatment will be ignored (treatment policy strategy); event-free patients at the end of the follow-up period will be censored and patients who were lost to follow up or dropped out of the trial will be censored at the last observation (hypothetical strategy).<br><br>Summary measure: Hazard ratio for the endpoint disease-free survival between the two treatment arms<br><br>**Secondary Endpoints**<br><br>• Complete remission rate (CRR), defined as the proportion of patients experiencing CR/CRi after induction therapy<br><br>• Relapse-free survival (RFS), defined as the time from achievement of CR/CRi after randomization to time of recurrence of the disease or death from any cause, whatever occurs first. Patients without an applicable event are censored on the last date of follow-up. [time frame: up to LPLV]<br><br>• Overall survival (OS), defined as the time from randomization to time of death from any cause. Patients without an applicable event are censored on the last date of follow-up. [time frame: up to LPLV]<br><br>• Patient-Reported Outcomes including Quality of Life:<br><br>o Health-related quality of life (QoL) is calculated as the new EORTC QLQ-C30 Summary Score recommended by the EORTC Quality of Life Group, which has been recently developed and evaluated. In addition, the EORTC QLQ function and symptom scores is calculated according to the actual EORTC Scoring Manual.<br><br>o Fatigue is calculated from the EORTC QLQ-FA12 according to the EORTC Scoring Manual.<br><br>o Sleep problems is calculated from the PSQI according to the corresponding scoring guidelines.	

			o Perceived cognitive impairments and impact of cognitive changes is calculated from the FACT-cog according to the corresponding scoring manual.<br><br>o Anxiety is calculated from the PHQ-4 according to the corresponding scoring manual [44].<br><br>o Depression is calculated from the PHQ-4 according to the corresponding scoring manual [44].<br><br>o Health state utilities are calculated based on the SF-36 generic instrument [45].<br><br>• Effectiveness of the investigational treatment is measured using the SF-36 generic instrument. A preference based single index is calculated using the SF-6D measure that facilitates obtaining health utilities and quality adjusted life years (QALYs).<br><br>• Health care resource utilization and costs are measured through the treatment course. Resource units and unit costs are collected separately by self-administered questionnaires at the end of each cycle and 3-monthly during maintenance therapy, as well as using relevant data from the eCRF and German reimbursement database.<br><br>**Analysis of the Primary Endpoints**<br><br>Since hypothesis tests are performed for both the short-term endpoint MRD-negativity and the long-term endpoint EFS, the null hypotheses H0ST and H0LT are tested using the Bonferroni-Holm approach in order to control the family-wise error rate in the strong sense. Hence, the smaller of the short-term and long-term p-values pST and pLT are tested at a two-sided significance level of α=0.025. In case the null hypothesis corresponding to the smaller p-value is rejected, the remaining null hypothesis corresponding to the larger p-value is tested at a two-sided significance level of α=0.05. In case the null hypothesis corresponding to the smaller p-value cannot be rejected, then the null hypothesis corresponding to the larger p-value has to be accepted as well.<br><br>The short-term primary endpoint MRD-negativity is analyzed using a generalized linear mixed model with the binary dependent variable “Patient MRD-negative (yes/no)”, including the fixed factors induction therapy (GO-147 vs. GO-1), ECOG PS (0 vs. 1-2), age (in years), sex, and the	

			random factor "center", using a variance components covariance matrix and residual log pseudo-likelihood as minimization criterion to fit the model. Due to the expected few number of patients and events per center, we chose to include "center" as a random factor in order to ensure stability for the statistical model based on the recommendation of Kahan & Harhay [49]. The short-term endpoint null hypothesis H0ST is tested based on the odds ratio of the factor induction therapy (GO-147 vs. GO-1). Missing values for the short-term primary endpoint MRD-negativity are replaced using multiple imputation by using of the fully conditional specification method [50], taking the variables, treatment group, age and ECOG PS into account. A complete-case analysis is done as a sensitivity analysis. Odds ratios are reported alongside with 97.5% and 95% confidence intervals, and a possible center effect is assessed by calculating the intra-class correlation coefficient and by presenting the results stratified for center.<br><br>The long-term primary endpoint EFS is analyzed using a Cox regression frailty model with the dependent variable EFS, including the fixed factors maintenance therapy (HiDAC+ glasdegib vs. HiDAC), induction therapy (GO-147 vs. GO-1), ECOG PS (0 vs. 1-2), age (in years), sex, and the random factor "center". Analogously to the short-term endpoint, a random-intercept model adjusting for "center" is used due to the expected few number of events per center. The long-term endpoint null hypothesis H0LT are tested by using the adjusted-degrees of freedom approach for frailty models proposed by Gray [51] which is implemented in the SAS procedure<br><br>PHREG. Dropout and loss-to-follow-up are treated as censoring events. Hazard ratios are reported alongside with 97.5% and 95% confidence intervals, and a possible center effect is assessed by calculating the intra-class correlation coefficient. EFS probabilities over time are displayed using survival estimates calculated using the Kaplan-Meier method.<br><br>Sensitivity analyses of the primary endpoints incorporate an analysis within the PP Population. Furthermore, the treatment effects are assessed descriptively within several subgroups of interest to identify potential prognostic and predictive factors. A sensitivity analysis of the long-term primary endpoint additionally includes the interaction between maintenance therapy and induction therapy.	

Statistics: additional analyses	#20b	Methods for any additional analyses (eg, subgroup and adjusted analyses)	**Analysis of the Secondary Endpoints**<br><br>Secondary time-to event endpoints are analyzed analogously to the primary short-term endpoint EFS by using cox regression frailty models adjusting for treatment, age, ECOG PS and center, determining hazard ratios with 95% confidence intervals and (descriptive) p-values. Furthermore, event probabilities over time are displayed using survival estimates calculated using the Kaplan-Meier method. The secondary endpoint complete remission rate (CRR) is analyzed analogously to the primary short-term endpoint, using generalized linear mixed models to estimate odds ratios with corresponding 95% confidence intervals and descriptive p-values.<br><br>**Safety Analysis**<br><br>The assessment of safety is based mainly on the frequency of adverse events (see Section 9) and on the number of laboratory values that fall outside of pre-determined ranges and/or show prominent worsening from baseline during the study phase. Adverse events are summarized by presenting the number and percentage of patients having any adverse events or serious adverse events, and having each individual type of adverse event, and by determining and summarizing the maximum individual toxicity grade (over all forms of toxicity) for each treatment cycle during the study phase. Furthermore, the most common AEs (those occurring in at least 10% of the treatment group) are determined. Any other information collected (e.g. severity or relatedness to study drug) are summarized as appropriate. Laboratory data are summarized by presenting summary statistics of raw data and changes from baseline values. Incidence rates are summarized along with two-sided Pearson-Clopper 95% confidence intervals and analyzed by (descriptive) chi-squared tests.<br><br>**Patient-Reported Outcomes (PROs) including Quality of Life Analysis (QoL)**<br><br>The QoL scales are scored and analyzed according to the EORTC recommendations as described in the EORTC QLQ-C30 scoring manual [52]. The Quality of Life subscales and single item sub-scores are summarized by the mean, standard deviation and median and plotted over time for all four treatment groups. The change from baseline in QoL until a respective time point is examined by means of a general linear mixed model adjusting for treatment group, ECOG PS (0 vs. 1-2), age	

			(in years) and sex as fixed factors and center as random factor, calculating least square means estimates and 95% confidence intervals. For details on analysis of patient-reported outcomes (PROs) see Section 10.2.2.<br><br>With regard to the analysis of data from SF-36 questionnaire see below (Health Economic Analysis).<br><br>**Health Economic Analysis**<br><br>Effectiveness of the investigational treatment is measured using the SF-36 instrument. Dimension scores and summary scores for physical and mental health are obtained and analyzed according to the SF-36 scoring manual [45] and summarized over time by mean, standard deviation and median for separate treatment arms. A preference based single index is<br><br>calculated using the SF-6D measure that facilitates obtaining health utilities for the use of cost-effectiveness analysis. Health care resource utilization data is summarized by mean, standard deviation and median for separate treatment arms. To calculate total costs, micro-costing approach is intended to be used. Health care resource utilization units are multiplied by German standard unit costs of the relevant resource items by patient.<br><br>Various clinical trial data are used to conceptualize and populate the cost-effectiveness model. QALYs are calculated according to state of the art health economic methodology using Kaplan-Meier curves of OS and RFS to determine the expected length of life and SF-36 scores to provide health state utilities (i.e. quality of life information) in the model. To extrapolate the data over the model time horizon, survival curves are fitted by treatment arms to OS and RFS data and the base case curve is selected on the basis of goodness of fit, if data quality permits. Health state utilities mapped from the EORTC QLQ-C30 instrument are used in scenario analysis. Frequency and severity of AEs are used to calculate AE treatment costs and disutilities. Health care resource utilization units and unit costs are used to calculate the expected cost through the patients´ treatment course, including medical costs, disease monitoring, hospitalizations, and potential other health care resources. Non-parametric, empirical distribution functions are built into the cost-effectiveness model to assess uncertainty around the model estimates, if data quality	

			permits. Furthermore, a mapping function between EORTC QLQ-C30 and SF-36 is generated and validated.	
Statistics: analysis population and missing data	#20c	Definition of analysis population relating to protocol non-adherence (eg, as randomised analysis), and any statistical methods to handle missing data (eg, multiple imputation)	Page 14, lines 396-398.<br><br>The Full Analysis Population includes all randomized patients with treatment groups assigned in accordance with the randomization, regardless of the treatment actually received. The analysis of data using the Full Analysis Population therefore follows the principles of Intention To Treat (ITT). This will be the primary analysis population for the primary and secondary efficacy endpoints.<br><br>If there are patients who were randomized but did not subsequently receive treatment, these are excluded from the Full Analysis Population for sensitivity analysis as they provide no information about efficacy or safety of the interventions under investigation. Then the analysis follows the modified ITT (m-ITT) principle [48].<br><br>**Per Protocol Population**<br><br>The Per Protocol (PP) Population comprises all patients of the Full Analysis Population without major protocol deviations. Definition of major protocol deviations is given in the statistical analysis plan (SAP). Analyses based on the PP Population serve as sensitivity analyses in order to assess the robustness of the results obtained from the Full Analysis Population.<br><br>**Safety Population**<br><br>The Safety Population is the primary population for the evaluation of treatment administration/compliance and all safety endpoints and comprises all patients enrolled who received at least one dose of study medication. Patients are analyzed according to the treatment actually received.<br><br>Missing values for the short-term endpoint MRD-negativity are replaced using multiple imputation (see Section 10.5.3 for details). For patients with incomplete follow-up, time to last follow-up date is used as the censoring time in the analysis of time-to-event data. Otherwise, no imputation of missing data is conducted.	

Methods: Monitoring				
Data monitoring: formal committee	#21a	Composition of data monitoring committee (DMC); summary of its role and reporting structure; statement of whether it is independent from the sponsor and competing interests; and reference to where further details about its charter can be found, if not in the protocol. Alternatively, an explanation of why a DMC is not needed	Pages 12 and 13, lines 343-453.<br><br>**Monitoring**<br><br>Monitoring is done by personal visits from a clinical monitor and by centralized monitoring according to the monitoring plan. The investigator must allow the monitor to verify all essential documents and must provide support at all times to the monitor. Monitoring is done in a risk-based manner.<br><br>By frequent communications (e-mails, letters, telephone, fax), the site monitor and the central monitor ensure that the trial is conducted according to the protocol and to regulatory requirements.<br><br>**Inspections / Audits**<br><br>Regulatory authorities and auditors authorized by the sponsor may request access to all source documents, the CRF, and other trial documentation. Direct access to these documents must be guaranteed by the investigator, who must provide support at all times for these activities.<br><br>**Data Monitoring Committee (DMC)**<br><br>A DMC is assembled. The DMC is composed of three independent experts, assessing the progress and safety data. The mission of the DMC is to ensure the ethical conduct of the trial and to protect the safety interests of patients in this trial.<br><br>The DMC meets virtually 3-monthly. Based on its review, the DMC provides the sponsor with recommendations regarding trial modification, continuation or termination.<br><br>Further details including DMC members are specified in the DMC charter.<br><br>The DMC charter is set up in accordance with applicable guidelines (EMEA/CHMP/EWP/5872/03 Corr, ICH Guidelines E3 E6, E9, Directive 2001/20/EC).	

Data monitoring: interim analysis	#21b	Description of any interim analyses and stopping guidelines, including who will have access to these interim results and make the final decision to terminate the trial	Page 13, lines 350-353.<br><br>Interim safety reports (DSURs) are prepared by the pharmacovigilance officer together with the Coordinating Investigator in accordance with legally required timeframes; data reconciliation is carried out where necessary and possible together with the data management based on already available CRF-AE data.	
Harms	#22	Plans for collecting, assessing, reporting, and managing solicited and spontaneously reported adverse events and other unintended effects of trial interventions or trial conduct	Page 13 , lines 343-353.<br><br>**Laboratory Safety Assessments**<br><br>Hematology, blood chemistry, coagulation and urinalysis assessments are drawn at the time points described in the Trial Schedule on page 13 and are analyzed by the site/Investigator at local laboratories. Laboratory certifications and normal ranges with units must be provided to the Coordinating Investigator.<br><br>If hematology (CBC with differentials) is obtained within 3 days of a scheduled blood draw, the collection needs not be repeated. For those patients achieving complete or partial hematological response, a CBC should be done at least 4 weeks after the BM assessment in order to confirm response. Hematology tests may be repeated also as clinically indicated.<br><br>If blood chemistry or coagulations are obtained within 3 days of a scheduled blood draw, the collection need not be repeated.	

			If a urinalysis was obtained within 2 days of the scheduled collection, it should not be repeated. For urinalysis, dipstick is acceptable. Microscopic analyses should be done in case of abnormal results (i.e., the presence of protein or blood).<br><br>**Adverse Events**<br><br>According to GCP, an adverse event (AE) is defined as follows: Any untoward medical occurrence in a patient administered a pharmaceutical product and which does not necessarily have a causal relationship with this treatment. An AE can therefore be any unfavorable and unintended sign (including an abnormal laboratory finding), symptom, or disease temporally associated with the use of a medicinal (investigational) product, whether or not related to the medicinal (investigational) product.<br><br>**An AE may be:**<br><br>• New symptoms/ medical conditions<br><br>• New diagnosis<br><br>• Changes of laboratory parameters<br><br>• Intercurrent diseases and accidents<br><br>• Worsening of medical conditions/ diseases existing before inclusion into the trial<br><br>• Recurrence of disease<br><br>• Increase of frequency or intensity of episodical diseases.<br><br>If an AE shows an undulating course of intensity, it must be reported only once per cycle, indicating the highest CTCAE (Version 5.0) grade. If an event stops and later restarts within the same cycle, all occurrences must be reported.<br><br>A pre-existing disease or symptom is not considered an AE unless there is an untoward change in its intensity, frequency or quality. This change is documented by an investigator.	

			Surgical procedures themselves are not AEs; they are therapeutic measures for conditions that require surgery. The condition for which the surgery is required may be an AE. Planned surgical measures permitted by the clinical trial protocol and the condition(s) leading to these measures are not AEs, if the condition leading to the measure was present prior to inclusion into the trial.<br><br>AEs are classified as "non-serious" or "serious".<br><br>The sponsor will keep detailed records of all adverse events reported by the investigators.<br><br>**Serious Adverse Event**<br><br>A serious adverse event (SAE) is one that at any dose:<br><br>• Results in death<br><br>• Is life-threatening (the term life-threatening refers to an event in which the patient was at risk of death at the time of event and not to an event which hypothetically might have caused death if it had been more severe)<br><br>• Requires patient hospitalization or prolongation of existing hospitalization<br><br>• Results in persistent or significant disability/ incapacity or<br><br>• Results in a congenital anomaly/ birth defect.<br><br>• Is medically significant (e.g. suspected transmission of an infectious agent via medicinal product). Moreover, there are other situations - such as important medical events that may not be immediately life threatening or result in death or hospitalization but may jeopardize the patient or may require intervention to prevent one of the other outcomes listed above.<br><br>**Expectedness**<br><br>An ‘unexpected’ adverse event is one the nature or severity of which is not consistent with the applicable product information (Reference Safety Information (RSI)), e.g. Investigator’s Brochure (IB), Summary of Product Characteristics (SmPC). Furthermore, reports which add significant	

			information on specificity or severity of a known adverse reaction are counted as 'unexpected' events.<br><br>**Suspected Unexpected Serious Adverse Reaction (SUSAR)**<br><br>SAEs that are both suspected, i.e. possibly related to IMP, and 'unexpected' for the respective IMP, i.e. the nature and/ or severity of which is not consistent with the applicable RSI are to be classified as Suspected Unexpected Serious Adverse Reactions (SUSARs).<br><br>All SUSARs are subject to an expedited reporting to the responsible ethics committee(s), the competent higher federal authority (i.e. BfArM), and to all participating investigators.	
Auditing	#23	Frequency and procedures for auditing trial conduct, if any, and whether the process will be independent from investigators and the sponsor	**Audits**<br><br>Regulatory authorities and auditors authorized by the sponsor may request access to all source documents, the CRF, and other trial documentation. Direct access to these documents must be guaranteed by the investigator, who must provide support at all times for these activities.	
**Ethics and dissemination**				
Research ethics approval	#24	Plans for seeking research ethics committee / institutional review board (REC / IRB) approval	Page 13, lines 364-370 and page 18, lines 499-520.<br><br>**Continuous Information to Independent Ethics Committee**<br><br>Pursuant to the German Drug Law (AMG) and the GCP Regulation, the EC and the competent higher federal authority are informed of all suspected unexpected serious unexpected adverse reactions (SUSARs) and all AEs resulting in death or being live-threatening, which occur during the trial. Both institutions are informed in case the benefit-risk assessment did change or any other new and significant hazards for patients' safety or welfare did occur. Furthermore, a report on all	

			observed serious adverse events (SAEs) is submitted once a year (Development Safety Update Report (DSUR)).<br><br>The EC and the regulatory authorities must be informed of the end of the trial. They have to be provided with a summary of trial results within one year after the end of the clinical phase (LPLV).	
Protocol amendments	#25	Plans for communicating important protocol modifications (eg, changes to eligibility criteria, outcomes, analyses) to relevant parties (eg, investigators, REC / IRBs, trial participants, trial registries, journals, regulators)	**Approval of Trial Protocol and Amendments**<br><br>Before the start of the trial, the trial protocol, informed consent document, and any other appropriate documents are submitted to the independent Ethics Committee (EC) as well as to the competent federal authority (BfArM). A written favorable vote of the EC and an (implicit) approval by the competent higher federal authority are a prerequisite for initiation of the clinical trial. The statement of EC should contain the title of the trial, the trial code, the trial site, and a list of reviewed documents. It must mention the date on which the decision was made and must be officially signed by a committee member.<br><br>Before the first patient is enrolled in the trial, all ethical and legal requirements must be met. All planned substantial changes (see §10, (1) of German GCP-Regulation) are to be submitted to EC and the competent federal authority in writing as amendments. They have to be approved by the EC and the competent federal authority.<br><br>The Coordinating Investigator or the NCT Trial Center, and if applicable the investigator(s) are keeping a record of all communication with the EC and the regulatory authorities.<br><br>The Coordinating Investigator should ensure that all persons assisting with the trial are adequately informed about the protocol and any amendments, the trial treatments, and their trial-related duties and functions.<br><br>The Coordinating Investigator should maintain a list of investigators and other appropriately qualified persons to whom he or she has delegated significant trial-related duties (Log of Staff).<br><br>The investigators should support monitoring, auditing and inspections.	

Consent or assent	#26a	Who will obtain informed consent or assent from potential trial participants or authorised surrogates, and how (see Item 32)	Page 9, line 237 and 238.<br><br>Before being admitted to the clinical trial, the participant must consent to participate after being fully informed by the investigator or a designated member of the investigating team about the nature, importance, risks and individual consequences of the clinical trial and their right to terminate the participation at any time.	
Consent or assent: ancillary studies	#26b	Additional consent provisions for collection and use of participant data and biological specimens in ancillary studies, if applicable	One of the exclusion criteria is the non-consent for biobanking and for registration, storage and processing of the individual disease-characteristics and course as well as information of the family physician about study participation. Furthermore, all investigators are highly encouraged to registers their AML-patients at the Study alliance Leukemia (SAL) registry, which facilitates access to further clinical AML-trials and collects structured follow-up data.	
Confidentiality	#27	How personal information about potential and enrolled participants will be collected, shared, and maintained in order to protect confidentiality	The data obtained in the course of the trial are treated pursuant to the General Data Protection Regulation (EU-DSGVO, EU 2016/679) and the Data Protection Law of the Federal State (Landesdatenschutzgesetz), and the § 40 (2a) AMG.<br><br>During the clinical trial, patients are identified solely by means of an individual identification code (Patient ID). Storage of trial findings on a computer is done in accordance with local data protection law and handled in strictest confidence. For protection of these data, organizational procedures are implemented to prevent distribution of data to unauthorized persons. The appropriate regulations of local data legislation are fulfilled in its entirety.	

		before, during, and after the trial	The patient consents in writing to relieve the investigator from his/her professional discretion in so far as to allow inspection of original data for monitoring purposes by health authorities and authorized persons (inspectors, monitors, auditors). Authorized persons may inspect the patient-related data collected during the trial, ensuring the data protection law.<br><br>The investigator has to maintain a patient identification list (Patient IDs with the corresponding patient names) to enable records to be identified.<br><br>Patients who did not consent to circulate their pseudonymized data must not be included into the trial.	
Declaration of interests	#28	Financial and other competing interests for principal investigators for the overall trial and each study site	Lines 526-530, page 18.<br><br>Before the start of the trial, the investigators disclose to the sponsor any proprietary or financial interests he or she might hold in the sponsors/ a funding company, in the investigational product(s), or any commercial organization being involved in the clinical trial. The investigator has also to confirm that he/she has not entered into any financial arrangement whereby the value of compensation paid could affect the outcome of the clinical trial.<br><br>The investigator agrees to update this information in case of significant changes.	
Data access	#29	Statement of who will have access to the final trial dataset, and disclosure of contractual agreements that limit such access for investigators	The investigator(s) archive all trial data (source data and Investigator Site File (ISF) including Patient Identification List and relevant correspondence) according to the Section 4.9 of the ICH Consolidated Guideline on GCP (E6) and to local law or regulations. The Patient Identification List is archived for at least 15 years after trial termination.	
Ancillary and post trial care	#30	Provisions, if any, for ancillary and	The period of treatment ends with the last visit of the sixth cycle of the maintenance therapy (EOT). After EOT patients are routinely followed-up and treated regarding standard of care	

		post-trial care, and for compensation to those who suffer harm from trial participation	according to the discretion of the treating physician. The period of observation (and the study) ends for all patients when the last patient being included and alive has been followed for at least 730 days (2 years) counted from this patient's day 1 (EOS).	
Dissemination policy: trial results	#31a	Plans for investigators and sponsor to communicate trial results to participants, healthcare professionals, the public, and other relevant groups (eg, via publication, reporting in results databases, or other data sharing arrangements), including any publication restrictions	The biostatistician prepares the final trial report together with the Coordinating Investigator within 12 months after the end of the study (database lock).<br><br>Interim safety reports (DSURs) are prepared by the pharmacovigilance officer together with the Coordinating Investigator in accordance with legally required timeframes; data reconciliation is carried out where necessary and possible together with the data management based on already available CRF-AE data.<br><br>All information concerning the trial is confidential before publication. Trial results will be published in peer-reviewed medical journals.	

Dissemination policy: authorship	#31b	Authorship eligibility guidelines and any intended use of professional writers	Authorship eligibility is based on the following criteria (ICMJE):<br>• Substantial contributions to the conception or design of the work;<br>or the acquisition, analysis, or interpretation of data for the work; AND<br>• Drafting the work or revising it critically for important intellectual content; AND<br>• Final approval of the version to be published; AND<br>• Agreement to be accountable for all aspects of the work in ensuring that questions related to the accuracy or integrity of any part of the work are appropriately investigated and resolved.<br>There is no intended use of professional writers.	
Dissemination policy: reproducible research	#31c	Plans, if any, for granting public access to the full protocol, participant-level dataset, and statistical code	After publication of the complete trial access to selected raw data is intended according to the applicable process.	
**Appendices**				
Informed consent materials	#32	Model consent form and other related documentation given to participants and authorised surrogates	German version only is attached as a supplementary document.	

Biological specimens	#33	Plans for collection, laboratory evaluation, and storage of biological specimens for genetic or molecular analysis in the current trial and for future use in ancillary studies, if applicable	Within the scope of this study biological samples are stored to develop further knowledge and understanding of diagnostic, prognostic and predictive markers present in AML patients, including their potential association with the study treatment. Pseudonymization of all samples is done in a two-step procedure, directly at sampling and when datasets are stored.<br><br>Responsibilities for storage of biological samples lie with the central molecular genetics laboratory of the University Hospital Heidelberg. Data ownership is and will remain with the University Hospital Heidelberg.<br><br>On the occasion of the informed consent procedure patients are explicitly informed about the arrangements for sample storage including their right to withdraw consent for further use of their biosamples at any time and that samples will be disposed in this case.	

It is strongly recommended that this checklist be read in conjunction with the SPIRIT 2013 Explanation & Elaboration for important clarification on the items. Amendments to the protocol should be tracked and dated. The SPIRIT checklist is copyrighted by the SPIRIT Group under the Creative Commons "Attribution-NonCommercial-NoDerivs 3.0 Unported" license. This checklist can be completed online using https://www.goodreports.org/, a tool made by the EQUATOR Network in collaboration with Penelope.ai

Trial Schedule

PHASE	BL	IT	IT	IT	IT	CT	CT	CT	CT	MT	MT	EOT	SA	FU	EOS
**DAY (OF CYCLE)**	-14-0	1	4,7	8-EOC	EOC[1]	1	2-3	4-EOC	EOC[2]	1-27	28/ EOC				
**Clinical assessments**															
Signs/symptoms	X				X				X		X^{3M}	X^{O}	X^{M}	X^{3M,Y}	X
Vital signs	X^{Height}	X	X	X^{W}	X	X^{O}		X^{W}	X		X	X^{O}	X^{M}	X^{3M,Y}	X
Physical examination	X	X^{O}	X	X^{W}	X	X^{O}		X^{W}	X		X	X^{O}	X^{M}	X^{3M,Y}	X
ECG	X	X^{O}				X^{O}			X		X	X^{O}			X
Extra medullary involvement	X				X^{HR}				X^{HR}		X$^{3M, HR}$	X^{O,HR}		X^{3M,Y,HR}	X^{HR}
Patient Reported Outcomes	X				X				X		X^{3M}	X^{O}		X^{3M,Y}	X
ECOG PS	X	X^{O}	X	X^{W}	X	X^{O}			X		X	X^{O}	X^{M}	X^{3M,Y}	X
**Laboratory assessments**															
Hematology	X	X	X	X^{W}	X	X^{O}		X^{W}	X		X	X^{O}	X^{M}	X^{3M,Y}	X
Basic blood chemistry	X	X	X	X^{W}	X	X^{O}		X^{W}	X		X	X^{O}	X^{M}	X^{3M,Y}	X
Extended blood chemistry & coagulation	X	X	X	X^{W}	X	X^{O}		X^{W}	X		X	X^{O}	X^{M}		
**Central laboratory assessments**															
Sample collection (BM, PB)	X			X^{15}	X				X		X^{3M}	X^{O}		X^{3M,Y}	X
MRD & Disease status	X				X				X		X^{3M}	X^{O}		X^{3M,Y}	X
**Health economic assessments**															
Resource utilization questionnaire					X				X		X^{3M}	X^{O}			
**Treatment**															
GO-147 (experimental arm)		X	X												
GO-1 (control arm)		X													
SOC: Chemotherapy		X$^{1-7}$	X$^{1-7}$			X^{C}	X^{C}								
Glasdegib / Placebo						X	X	X^{D28}		X	X				
Drug Compliance									X		X	X^{O}			
**Safety**															
Concomitant medications & treatment	X	X$^{1-7}$	X$^{1-7}$	X^{W}	X	X	X	X^{W}	X		X	X			
AE assessment		X$^{1-7}$	X$^{1-7}$	X^{W}	X	X	X	X^{W}	X		X	X	X^{M}		
Pregnancy test (WOCBP only)	X	X^{O}				X			X^{2C}		X	X^{O}	X^{M}		
**Screening and Baseline**															
Informed consent	X														
Demographics	X														
Medical/oncologic history	X														
Genetic assessment (central lab)	X														
Cytogenetics	X														
ECHO	X														
Abdominal ultrasound	X														
Urinalysis	X														
Virus diagnostics	X														
Enrollment & Randomization	X														

[1] includes treatment-free (except conditional salvage therapy) recovery period of 3-5 weeks, [2] includes treatment-free recovery period of up to 2 weeks if needed

Abbreviations used in the table:

- **1-7** **Days 1 to 7** (7 days Cytarabine, 3 days Daunorubicine)
- **2C** Only in **2nd cycle CT**
- **3M** To be done **3-monthly**
- **15** At day 15 of IT
- **BL** Baseline (within 14 days)
- **C** Cytarabine only
- **CT** Consolidation therapy (2 treatment cycles and subsequent treatment-free recovery period if needed).
- **D28** Stop at cycle day 28
- **EOC** End of cycle
- **EOS** End of Study (for all patients: 2 years after LPFV)
- **EOT** End of treatment (Within 7 days after or on Last Visit MT)
- **FU** **Observational follow-up** (3-monthly starting from Last Visit MT until EOS)
- **Height** At baseline incl. height in cm
- **HR** Post baseline only in patients with complete or partial hematological response
- **IT** Induction therapy (1 treatment cycle á 7 days and subsequent treatment-free recovery period)
- **M** To be done **monthly**
- **MT** Maintenance therapy (6 cycles)
- **O** To be **omitted** if done within preceding 48 hours
- **SA** Safety Follow-up (8 weeks after EOT)
- **W** To be done in **weekly intervals** (preferably same day per week)
- **Y** After 2 years from study day 1, on-site visits are no longer mandatory and may be replaced by contacting the treating physician or mailing the questionnaire. In this case, no further samples are collected.

Further descriptions of the study phases, number of visits and exact days are given in appendix 19.4.
